# Integrating network pharmacology analysis and pharmacodynamic evaluation for exploring the active components and molecular mechanism of moutan seed coat extract to improve cognitive impairment

**DOI:** 10.3389/fphar.2022.952876

**Published:** 2022-08-12

**Authors:** Yue Wang, Xinyan Wu, Kailin Yang, Qing Liu, Baoping Jiang, Runmei Yang, Peigen Xiao, Chunnian He

**Affiliations:** Key Laboratory of Bioactive Substances and Resources Utilization of Chinese Herbal Medicine, Ministry of Education, Institute of Medicinal Plant Development, Chinese Academy of Medical Science, Peking Union Medical College, Beijing, China

**Keywords:** alzheimer’s disease, high-throughput sequencing, molecular docking, seed coat extracts of Paeonia suffruticosa, suffruticosol B, cholinergic function, antioxidant, anti-inflammatory

## Abstract

*Paeonia suffruticosa* (Moutan) is a traditional medicinal plant in China. Its seed coat is rich in resveratrol oligomer, especially suffruticosol B (SB). Previous studies had shown that the seed coat extracts of *Paeonia suffruticosa* (PSCE) had good cholinesterase inhibitory activity and neuroprotective effect, but the effective dose range was unknown, and the pharmacodynamic components and molecular mechanism of PSCE had not been discussed. The current study aimed to screen the pharmacodynamic components in PSCE and investigate the improvement effect of PSCE and the selected SB on scopolamine-induced cognitive dysfunction in mice and its mechanism. The results of high-throughput sequencing and bioinformatics analysis showed that suffruticosol B (SB) and *trans*-gnetin H (GH) might be the main active components of PSCE; PSCE might improve cognitive dysfunction through p53, HIF-1, MAPK, and PI3K-Akt signaling pathways, while SB and GH might improve cognitive dysfunction through HIF-1 signaling pathway. SB and GH had good molecular docking activity with the target of HIF-1 signaling pathway. The pharmacodynamic activities of PSCE and SB were further verified by behavioral experiments. PSCE and SB could improve the recognition ability of familiar and new objects and shorten the escape latency in the Morris Water Maze test (PSCE 120 mg∙kg-1, *p* < 0.05; SB 60 mg∙kg-1, *p* < 0.01); PSCE and SB could increase Ach and GSH levels, enhance the activities of ChAT, SOD and CAT, decrease the levels of IL-1β, IL-6, and TNF-α, and decrease the activity of AChE. In conclusion, the results indicated that PSCE might exert pharmacodynamic activity through multiple components, targets, and pathways, and SB and GH might be the main active components of PSCE. PSCE and SB might improve cognitive dysfunction by regulating cholinergic, antioxidant, and anti-inflammatory effects. These results indicated that PSCE and SB might be potential anti-AD drug candidates, providing a scientific basis for the development and utilization of Moutan bark.

## Introduction

Alzheimer’s disease (AD) is a neurodegenerative disease mainly characterized by memory and cognitive dysfunction. The main pathological features are the appearance of neurofibrillary tangles (NFTs) and senile plaques (SPs) and the loss of cholinergic neurons (Kong et al., 2020). The latest data shows that more than 50 million people with dementia worldwide will reach 152 million by 2050 (Barnett, 2019), placing a severe burden on patients, families, and society. Due to the complex pathogenesis of AD, currently, there are only six drugs approved by the FDA for the treatment of AD, and these drugs only target the symptoms but cannot slow down or prevent the progression of the disease (Alexander et al., 2021; Alzheimer’s, 2022). Therefore, there is an urgent need to find new safe and effective drugs to treat AD.

Natural products have attracted attention due to their comprehensive sources and advantages in improving clinical quality of life. Natural products also contain abundant anti-AD active components (Figueiró et al., 2010), such as resveratrol and its oligomers. Resveratrol can improve patients’ symptoms by regulating levels of Aβ, anti-oxidant enzymes and inflammatory factors (Liu et al., 2015; Martin, 2017; Sawda et al., 2017; Rao et al., 2020), while resveratrol oligomers have significant anti-tumor (He et al., 2019; Meneses-Gutierrez et al., 2019; Aja et al., 2020), anti-inflammatory (Milanezi et al., 2019; Nagumo et al., 2019; Cheng et al., 2020), antioxidant (Meneses-Gutierrez et al., 2019; Shang et al., 2022; Yang et al., 2022), hypoglycemic (Liu et al., 2021; Wang et al., 2022) and neuroprotective effects (Jeon et al., 2007; Moussa et al., 2017; Liu et al., 2020) and are generally superior to resveratrol. Moutan is a small decedent shrub of the *genus Paeonia*. As a traditional therapeutic part, its root bark has the effect of clearing heat and cooling blood, promoting blood circulation, and removing blood stasis (Commission, 2020). Its seeds can be prepared into oil, which has benefits such as maintaining brain and cardiovascular health (Qianhao et al., 2021). Before pressing seed oil, the seed coat of Moutan will be removed, accounting for about 1/3 of the seed quality. Most of them are treated as garbage, which greatly wastes the resources of traditional Chinese medicine. Previous studies in our laboratory found the seed coats of Moutan had abundant stilbenes, especially resveratrol oligomers (Chunnian et al., 2016; Liu et al., 2020). These oligomers could inhibit cholinesterase activity and protect OGD/R damaged PC12 cells *in vitro*. In addition, the seed coat extracts of *Paeonia suffruticosa* (PSCE, 150 and 600 mg·kg-1) could improve scopolamine-induced learning and memory impairment in mice, improve cholinergic injury, and improve the antioxidant and anti-inflammatory ability of brain tissues (Liu et al., 2020), indicating that PSCE has specific anti-AD potential. Furthermore, SB isolated from PSCE had stronger β-secretion *in vitro* and cholinesterase inhibitory activity (Choi et al., 2011; Liu et al., 2020). However, the safe and effective dose range and the molecular mechanism of PSCE were unknown, and the components exerting pharmacodynamic activity were not explored *in vivo*.

High-throughput screening (HTS2) is a pathway-centered, high-throughput drug discovery technology using transcriptome high-throughput sequencing technology (Li et al., 2012), widely used in pharmacological treatment activity studies due to its higher sensitivity and high-throughput characteristics.

Therefore, to investigate the mechanism of PSCE at the cellular level and to screen the main components, this study will be conducted using high-throughput sequencing technology and bioinformatics analysis methods such as molecular docking. Furthermore, to seek safe and effective AD prevention drugs and strengthen the development and utilization of non-medicinal parts of Moutan, this study will investigate the improvement effect of PSCE and the screened SB on scopolamine-induced cognitive impairment in mice, and further explore the underlying mechanisms regulating cholinergic activity, oxidative stress and neuroinflammation. The present study provides support for potentially more effective treatment of AD.

## Materials and methods

### Materials and reagents

PSCE was prepared by our laboratory group. The preparation method comprised the following steps: the dried seed shells were pulverized and extracted with 70% ethyl alcohol (EtOH) by soaking at room temperature for 24 h twice. The combined EtOH extract was concentrated under reduced pressure, and drying the extracts were in a vacuum. The seed coats of *P. suffruticosa* were obtained from Heze, Shandong Province, P. R. China, and identified by Professor Chunnian He. A voucher specimen (2017001) has been deposited in the Seed Resource Bank of the Institute of Medicinal Plant Development, Chinese Academy of Medical Sciences and Peking Union Medical College, Beijing, P. R. China.

Ten stilbenes were isolated, purified, and identified from PSCE in our laboratory using a previously described method (He et al., 2010; He C.-N. et al., 2013; He C. et al., 2013).

### High-throughput sequencing and bioinformatic analysis

#### Cell culture and drug treatment

Hepatocellular carcinoma cells (HepG2) were cultured in Dulbecco’s Modified Eagle Medium (DMEM, HyClone Laboratories Inc., United States) supplemented with 10% fetal bovine serum (FBS, Gibco Laboratories Inc., United States) and 1% Penicillin/Streptomycin (Gibco Laboratories Inc., United States) at 37 °C in a humidified incubator containing 5% CO_2_. The logarithm growth cells were trypsinized by trypsin (Gibco Laboratories Inc., United States), collected, and then inoculated in a 384-well culture plate (3,000 cells per well). After 24 h of culture, the medium was discarded and replaced with the fresh medium containing PSCE 0.007 mg·mL-1 and 10 stilbenes 0.005 mg·mL-1, and the cells were cultured for another 24 h.

#### RNA extraction, library construction, and sequencing

After drug exposure, the cells were collected and washed twice with PBS to remove the drug. Total RNA was extracted using TRIzol reagent (Thermo Fisher Technologic Inc., USA) according to the kits’ instructions. The concentration and purity of total RNAs were measured by spectrophotometry. The RNA samples were sent to the Wang Dong research Group of Tsinghua University for library construction. Then the Illumina HiSeq 2,500 platform was used for high-throughput sequencing, referring to HTS2 experimental procedures (Hairi et al., 2012; Li et al., 2012).

#### Bioinformatics

The regular protocol preprocessed the raw data. DESeq was used to identify the differentially expressed genes (DEGs). DEGs were set using the following standard: |Log2(Fold Change)|≥0.5 and *p* ***-*** value < 0.05.

The GO functional and KEGG signaling pathway enrichment analysis of the DEGs were performed with R studio (https://www.r-project.org/) to identify the biological functions and pathways, respectively. Disease enrichment analysis based on GAD database with DAVID 6.8 (https://david.ncifcrf.gov/home.jsp) Statistical significance was set at *p* < 0.05.

Based on the above enrichment analysis and the content of 10 stilbenes in PSCE, this study identified the main components in PSCE and key signaling pathways for further molecular docking verification.

#### Molecular docking

Molecular docking techniques can dock small molecules into the protein binding site (Gandhi et al., 2019). After screening, the main components SB and GH were selected as ligand molecules and docked with the target protein receptor of DEGs enriched in the key HIF-1 signaling pathway, respectively. The 3D structures of SB and GH were acquired from the Pubchem Compound database (https://pubchem.ncbi.nlm.nih.gov/). The X-ray crystal structures of IL6, EIF4EBP1, ENO2, ERBB2, EDN1, HIF1A, HK2, and VEGFA were obtained from the Protein Data Bank (https://www.rcsb.org/). Molecular docking was performed by AutoDock Vina and AutoDock Tools (The Scripps Research Institute, La Jolla, CA, United States), and PyMOL (Schrödinger, Inc, New York, NY, United States) was used for visual processing. The amino acid residues in the hydrogen-bonded receptor protein to the compound molecule were marked. Binding Energy was used as a reference for docking results. If the docking energy value was less than -5.0 kcal·mol-1, it indicated good binding activity (Carmelo-Luna et al., 2021).

### Animals and experimental groups

84 male C57BL/6 mice (2 months) were purchased from Sibeifu Biotechnology Co. Ltd. (Beijing, China). Experimental animals were provided free access to food and water at a constant temperature (25 ± 2°C) and humidity (55 ± 10%) on a 12 h light/dark cycle. This research was approved by the Committee on Care and Use of Laboratory Animals of the Institute of Medicinal Plant Development, Chinese Academy of Medical Sciences.

**FIGURE 1:**
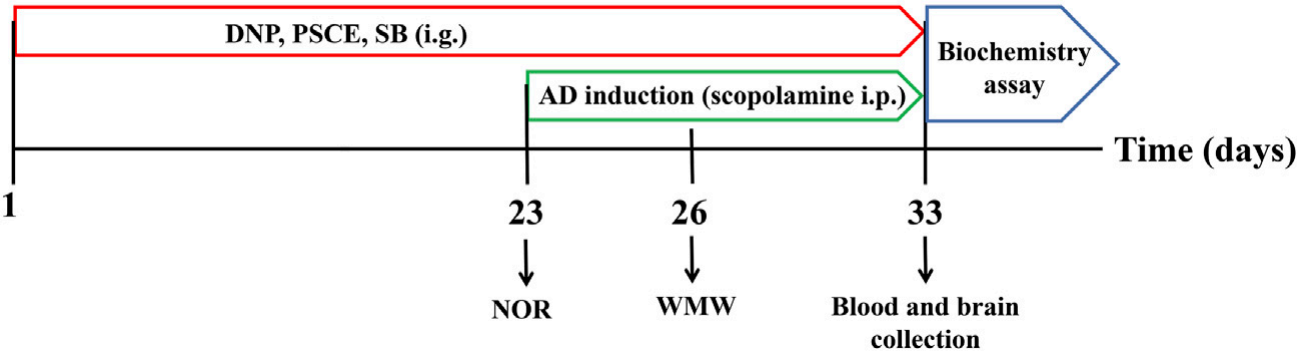
Schematic diagram of the experimental and treatment designs of mice.

Animals were randomly divided into seven groups (12 rats in each): Control group (sterile water), Model group (sterile water), DNP group (donepezil, 3 mg∙kg-1, Shanghai Aladdin Biochemical Technology Co., Ltd, Shanghai, China), PSCE group (30 and 120 mg∙kg-1). SB group (15 and 60 mg∙kg-1). The mice were orally administered the corresponding solutions for 22 days. Except Control group received normal saline, all mice were intraperitoneally injected with scopolamine (1.5 mg∙kg-1, Shanghai Aladdin Biochemical Technology Co., Ltd, Shanghai, China) once 30 min before the behavioral tests, and the experimental process is shown in Figure 1.

### Behavioral tests

#### New object recognition

NOR is widely used to assess short-term and non-spatial learning and memory in rodents (Cocco et al., 2020). The experimental process included the adaptation, familiar, and test stages. During the adaptation period, the mice were placed in a white box (40 cm × 40 cm × 40 cm) for 5 min. During the familiar stage, two identical objects were put into the box, and the distance between the two objects and the wall was kept the same. The mice were allowed to explore freely for 5min. One of the familiar objects was transformed into a new object during the test stage. The mice were allowed again to explore for 5 min. The video tracking system SuperMaze automatically recorded the mice’s time exploring familiar and new objects. The discrimination index (DI) evaluated the animals’ learning and memory abilities.

The discrimination index (DI) was calculated according to the following equation: DI = (time spent on the new object - time spent on the familiar object)/(time spent on the new object - time spent on the familiar object).

#### Morris water maze

MWM is used to assess rodents’ spatial learning and memory (Zhang et al., 2016). The device consisted of a white circular pool (diameter:100 cm; height: 50 cm) and white transparent platform (diameter: 9 cm; height:15 cm). The pool was divided into four quadrants (SW, NE, SE, and NW). The platform was located 1 cm underwater in the center of one quadrant and remained unchanged throughout the training period. The experiment process included the acquisition training and probe trial. During the acquisition training, the mice were allowed to search for the hidden platform for 60 s. If the mice did not find the platform, they would be guided to seek the hidden platform and stay on it for 20 s. The acquisition training was conducted two trials per day for five consecutive days. During the probe trial, the platform was removed, and the mice were allowed to swim for 60 s. The video tracking system SuperMaze automatically recorded the escape latency and the number of times the animal crossed the target quadrant and platform.

### Biochemical assay

After the behavioral tests described above, all mice received intraperitoneal anesthesia using tribromoethanol 200 mg/kg (Shanghai Maclin Biochemical Technology Co., Ltd, Shanghai, China). Blood was collected from the mice using the eyeball removal method, and serum was separated from the blood. The mice were sacrificed by decapitation, and the brain tissue was stripped out quickly. A BCA protein assay kit was used to assess protein concentration. Then, the activities of AChE, ChAT, SOD, and CAT and the levels of Ach and GSH in the brain were measured utilizing kits (Nanjing Jiancheng Bioengineering Institute, Nanjing, China) according to the instructions of the kits. The levels of IL-1β, IL-6, and TNF-α in serum were determined using ELISA kits (Beijing HuaYing Institute of Biological Technology, Beijing, China).

### Statistical analysis

All data were analyzed using the SPSS 22.0 software (IBM, Endicott, NY) and expressed as means ± SEM; the differences between the groups were considered statistically significant if *p* was <0.05. In the acquisition training of MWM, the escape latency was analyzed by repeated measure analysis of variance (ANOVA). All other data were statistically analyzed by one-way analysis of variance (ANOVA). If the results showed equal variances, the LSD test was used; if not, the Tamhane T2 (M) test was used.

## Results

### High throughput sequencing analysis in HepG2 cells between drug-treated and untreated groups

#### PSCE and 10 stilbenes treatment induced changes in gene expression in HepG2 cells

**FIGURE 2:**
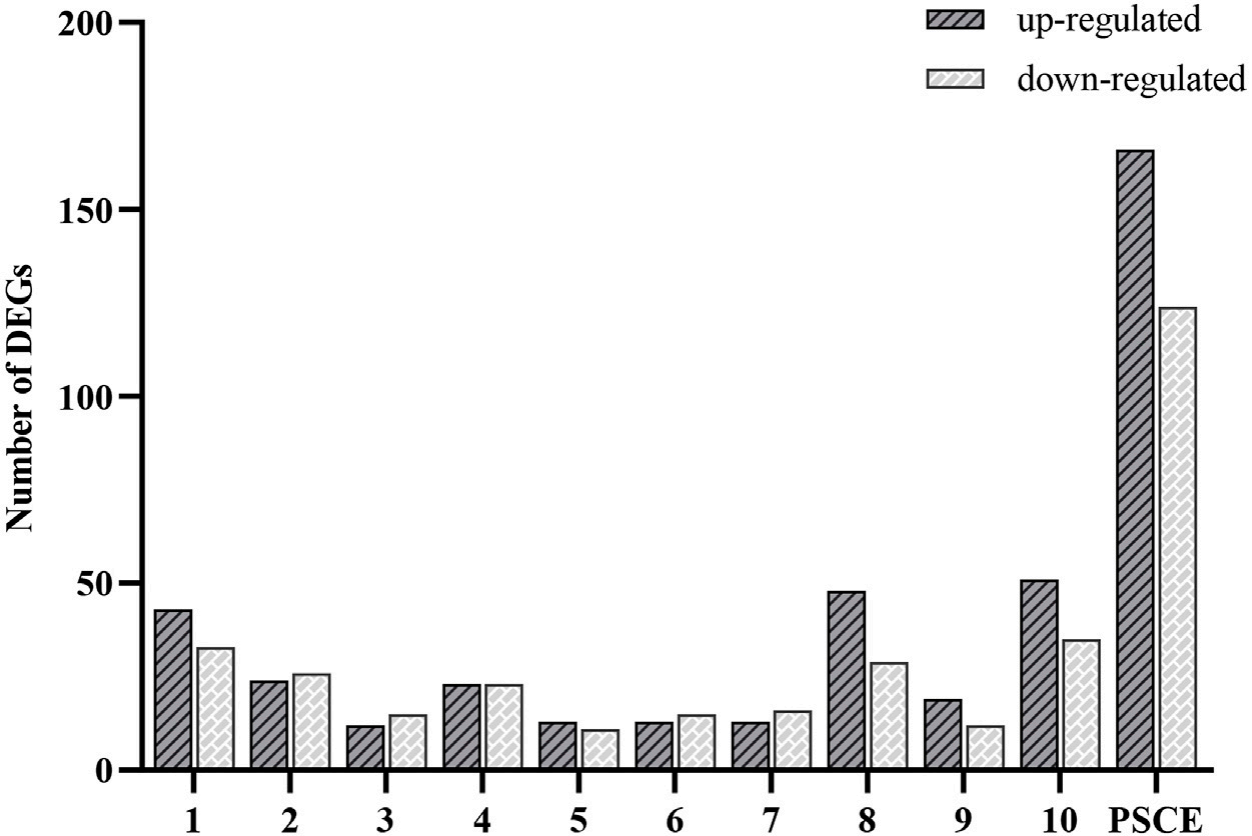
Analysis of DEGs between drug-treated and untreated groups (n = 4 per group). Abscissa: different drugs, including 10 stilbenes (1. suffruticosol A; 2. suffruticosol B; 3. suffruticosol C; 4. *trans*-resveratrol; 5. *cis-ε-*viniferin; 6. *trans-ε*-viniferin; 7. *cis*-suffruticosol D; 8. *cis*-gnetin H; 9. *trans*-suffruticosol D; 10. *trans*-gnetin H) and PSCE (the seed coat extracts of Moutan), ordinate: number of the up-regulated and down-regulated DEGs. The length of the column indicated the number of DEGs in this drug.

We used the high-throughput sequencing analysis to identify the DEGs in HepG2 cells after PSCE and stilbenes treatment. 166 DEGs were up-regulated, and 124 were down-regulated after PSCE treatment. Results of stilbenes treatment groups were established in Figure 2.

#### The GO functional enrichment analysis of DEGs altered by PSCE and 10 stilbenes

The GO functional enrichment analysis revealed that DEGs on PSCE and 10 stilbenes were enriched in the biological process (BP), molecular function (MF), and cellular component (CC).

**FIGURE 3:**
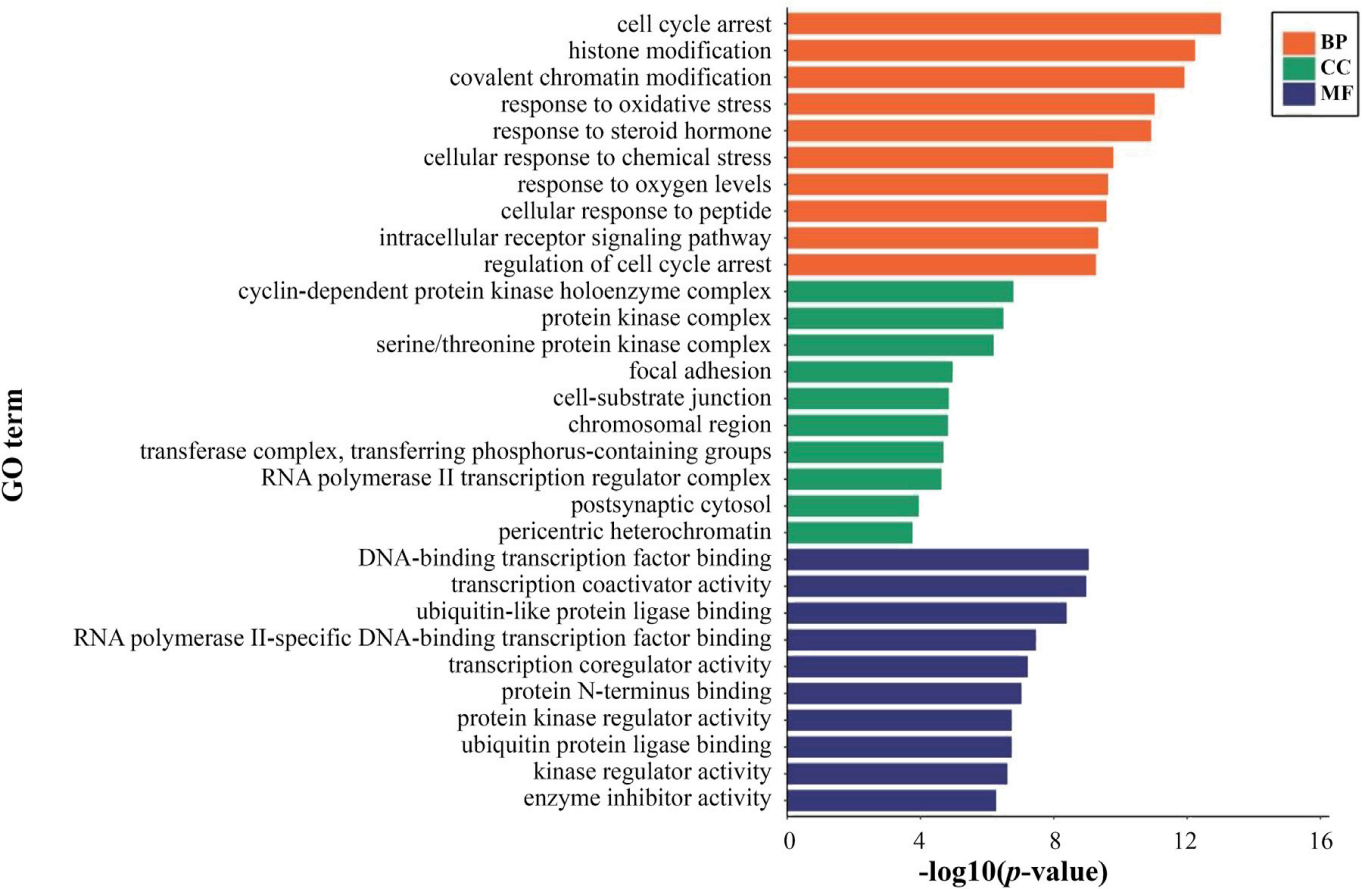
The top 10 significant terms of GO functional enrichment analysis of DEGs on PSCE. Abscissa: log (*p*-value), ordinate: GO terms, including biological process (orange), molecular function (green), cellular component (blue). The length of the column indicated the significant -log (*p*-value) of this GO term. The larger the -log (*p*-value) was, the more significant the enrichment was.

1894 GO items of DEGs on PSCE were screened. The biological processes of DEGs were significantly enriched in cell cycle arrest, histone modification, etc. The cellular components of DEGs were significantly enriched in cyclin-dependent protein kinase holoenzyme complex, protein kinase complex, etc. The molecular functions of DEGs were significantly enriched in DNA-binding transcription factor binding, transcription coactivator activity, etc. The top 10 enriched GO terms among BP, CC, and MF were presented in Figure 3.

10 stilbenes mainly participated in the regulation of intracellular enzymes and hormones, signal transduction, cell adhesion, cell proliferation and apoptosis, as well as biological processes related to angiogenesis, and possessed the molecular functions of regulating enzyme binding, hormone binding and transcription factor binding, as well as activity of transcription factors and enzyme inhibitors. It is closely related to all parts of cells, mainly located in intracellular complexes, cell junctions, fibers, synapses, cell membranes and nuclear membranes, etc. The top 10 enriched GO items of DEGs were enriched in Supplementary Figure S1.

#### The KEGG pathway enrichment analysis of DEGs altered by PSCE and 10 stilbenes

**FIGURE 4:**
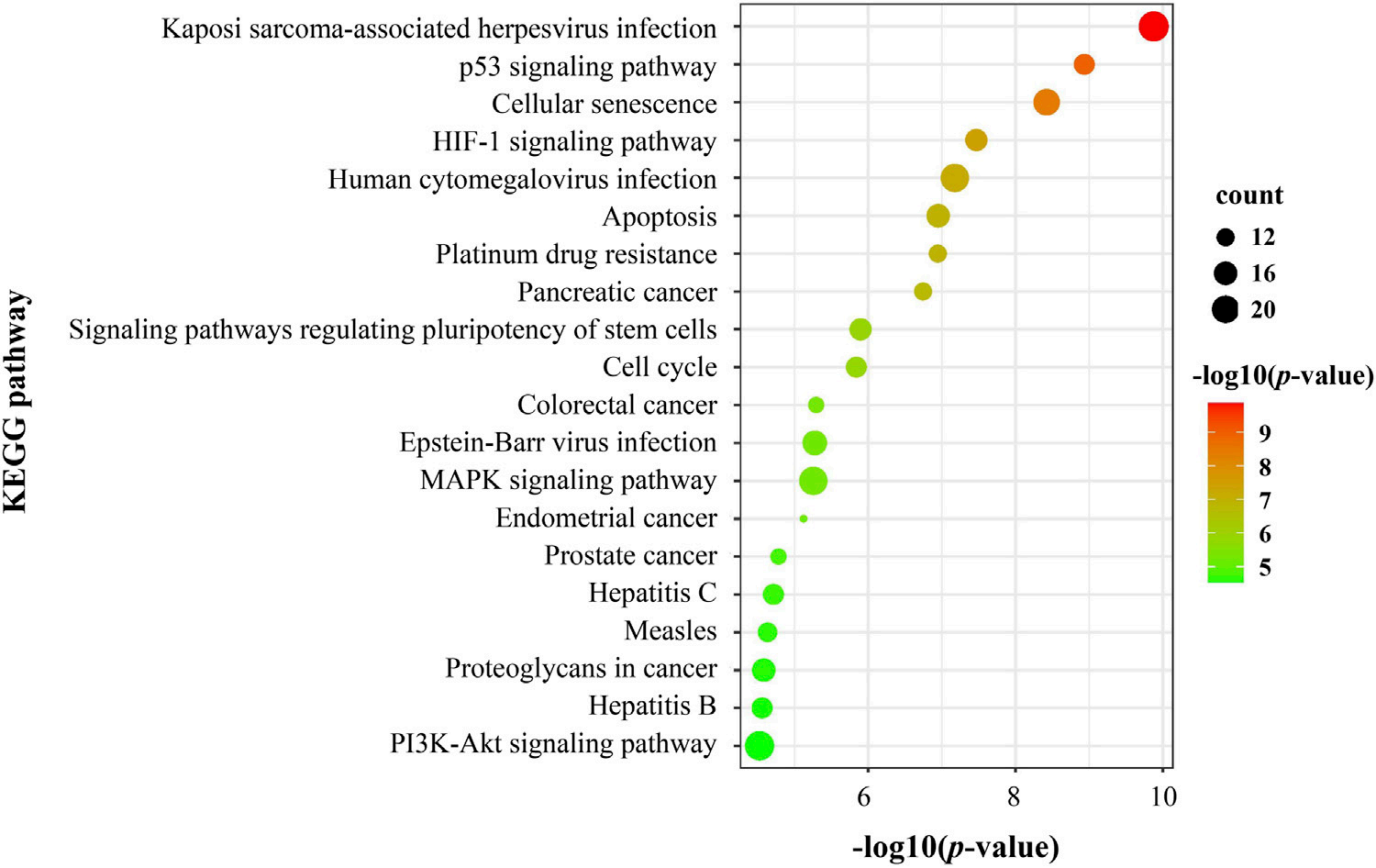
The top 20 significant terms of KEGG pathway enrichment analysis of DEGs on PSCE. Abscissa: log (*p*-value), ordinate: KEGG pathway. The size of the dot indicated the number of DEGs enriched in the pathway. The color indicates the significant -log (*p*-value) of this pathway. The larger the -log (*p*-value) was, the more significant the enrichment was.

The KEGG pathway enrichment analysis of DEGs on PSCE was shown that 103 signaling pathways were enriched, including p53, HIF-1, MAPK, and PI3K/Akt signaling pathway, etc. The top 20 pathways were visualized in Figure 4.

The KEGG pathway enrichment analysis of DEGs on 10 stilbenes was mainly enriched in HIF-1, ErbB, PI3K/Akt signaling pathways, etc. (Table 1) The details were presented in Supplementary Figure S2. The results revealed that the signaling pathways enriched of DEGs on PSCE were mainly related to compounds **1**, **2**, **5**, **6**, **7**, **8**, **9**, and **10**.

**TABLE 1:** The main KEGG pathways enriched by DEGs on 10 stilbenes.

Number	Compound	The main KEGG pathway
1	suffruticosol A	ErbB, PI3K/Akt, HIF-1, Wnt signaling pathways
2	suffruticosol B	HIF-1 and ErbB signaling pathways
3	suffruticosol C	ErbB and C-type lectin receptor signaling pathways
4	*trans*-resveratrol	Sphingolipid signaling pathway
5	*cis-ε*-viniferin	AMPK and PI3K/Akt signaling pathways
6	*trans-ε*-viniferin	HIF-1, p53, and PI3K/Akt signaling pathways
7	*cis*-suffruticosol D	HIF-1, TNF, and ErbB signaling pathways
8	*cis*-gnetin H	ErbB and HIF-1 signaling pathways
9	*trans*-suffruticosol D	MAPK signaling pathway
10	*trans*-gnetin H	HIF-1 and RIG-I-like receptor signaling pathways

#### The disease enrichment analysis of DEGs altered by PSCE and 10 stilbenes

**FIGURE 5:**
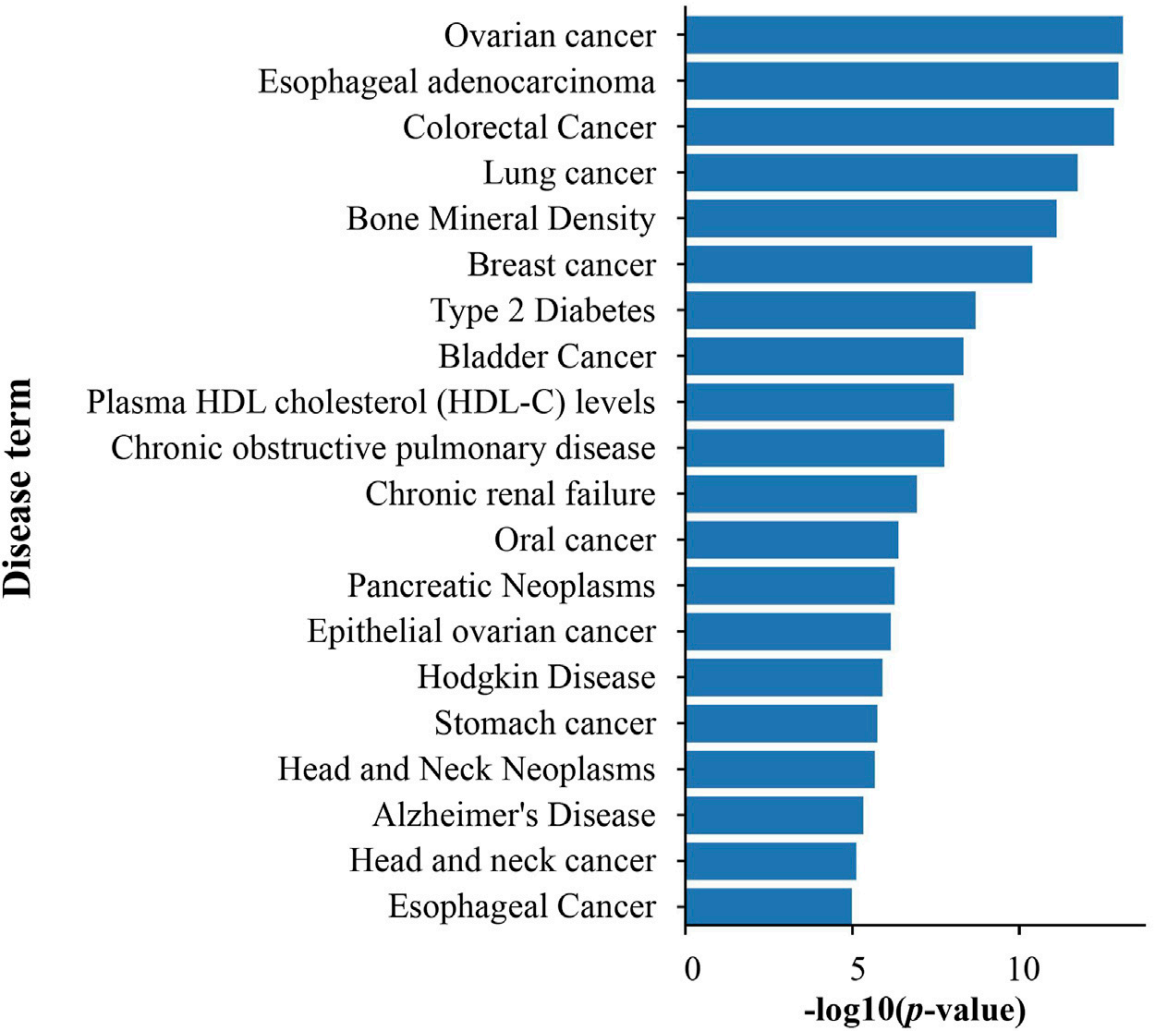
The top 20 disease enrichment analysis of DEGs on PSCE. Abscissa: log (*p*-value), ordinate: disease terms. The length of the column indicated the significant -log (*p*-value) of this disease term. The larger the -log (*p*-value) was, the more significant the enrichment was.

Based on the GAD database, diseases of DEGs on PSCE were significantly enriched to cancer (ovarian cancer, colorectal cancer), neurodegenerative diseases (Alzheimer’s disease), metabolic diseases (type 2 diabetes, obesity), etc. The top 20 enriched disease terms were visualized in Figure 5.

Meanwhile, DEGs of 10 stilbenes were significantly enriched in cancer (breast cancer, colorectal cancer), mental disorders (Schizophrenia, Alzheimer’s disease), metabolic diseases (type 2 diabetes, obesity), asthma, etc. The details were showed in Supplementary Figure S3. The results showed that stilbenes enriched in terms of neurodegenerative diseases included compounds **2**, **4**, **5**, **7**, **8**, and **10**.

#### SB and GH had good docking activity with the target proteins in HIF-1 signaling pathway

**FIGURE 6:**
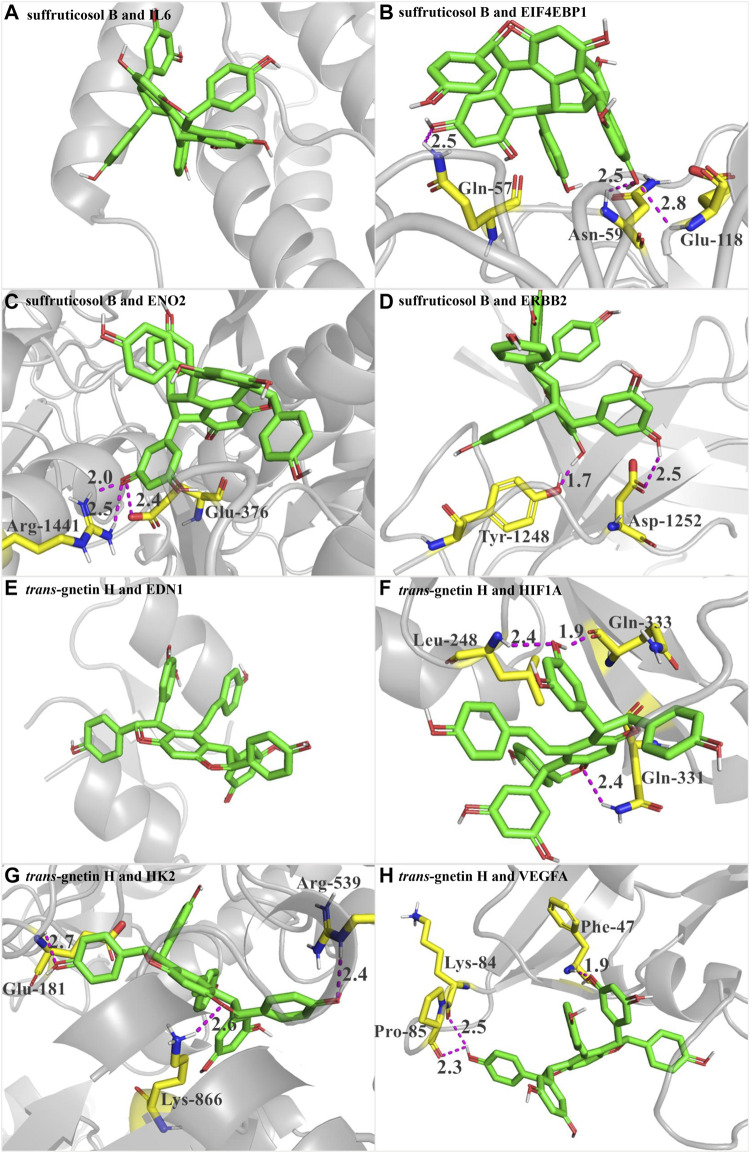
Interaction between SB and GH and target proteins in HIF-1 signaling pathway using molecular docking analysis. **(A)** the docking between suffruticosol B (SB) and IL6. **(B)** the docking between SB and EIF4EBP1. **(C)** the docking between SB and RNO2. **(D)** the docking between SB and ERBB2. **(E)** the docking between trans-gnetin H **(GH)** and EDN1. **(F)** the docking between GH and HIF1A. **(G)** the docking between GH and HK2. **(H)** the docking between GH and VEGFA. Took B for example, Figure B shows the docking between SB and EIF4EBP1. The green-based small molecule was compound SB, and the yellow-based molecule was the amino acid residue of EIF4EBP1 (Gln-57, Asn-59, Glu-118). Purple dotted line represented hydrogen bonding between SB and EIF4EBP1. SB formed hydrogen bonds with EIF4EBP1 through Gln-57, Asn-59, and Glu-118.

In PSCE, the compounds with higher content were compounds **1**, **2**, **3**, **6**, **9**, and **10** (Liu et al., 2020). Combined with the results of the KEGG signaling pathway and disease enrichment, SB and GH might be the main components of PSCE. Therefore, the target proteins of DEGs enriched in the critical HIF-1 signaling pathway were docked with SB and GH, respectively. The docking binding affinity was less than -5.0 kcal·mol-1 in Table 2, which indicated good binding activity between compound and target protein. The docking results were visualized as shown in Figure 6. SB did not establish hydrogen bond connections with IL6, and the same was true of the combination between GH and EDN1, which might dock by other bonds. The other target proteins were able to bind with SB and GH in a hydrogen bond. Such as SB, it could form hydrogen bonds with EIF4EBP1 through Gln-57, Asn-59, and Glu-118.

**TABLE 2:** The molecular docking affinity and binding sites of SB and GH with target proteins in HIF-1 signaling pathway.

Compound	Target protein	affinity (kcal·mol-1)	Binding site
suffruticosol B	IL6	-7.1	—
	EIF4EBP1	-10.1	Gln-57, Asn-59, Glu-118
	ENO2	-6.6	Arg-1441, Gln-376
	ERBB2	-7.5	Tyr-1248, Asp-1252
*trans*-gnetin H	EDN1	-5.8	—
	HIF1A	-7.2	Leu-248, Gln-333, Gln-331
	HK2	-8.9	Glu-181, Arg-539, Lys-866
	VEGFA	-8	Lys-84, Pro-85, Phe-47

### PSCE and SB improved scopolamine-induced spatial and non-spatial learning and memory impairment in mice

Through high-throughput sequencing screening, we speculated that SB and GH were the key active components in PSCE. Combined with previous studies that SB had stronger β-secretion *in vitro* and cholinesterase inhibitory activity (Choi et al., 2011; Liu et al., 2020; Tian et al., 2020), so we selected SB as the present research object to preliminarily explore its effect and mechanism of improving scopolamine-induced cognitive impairment.

#### PSCE and SB improved the mice’s ability to discriminate between new and familiar objects in the NOR

**FIGURE 7:**
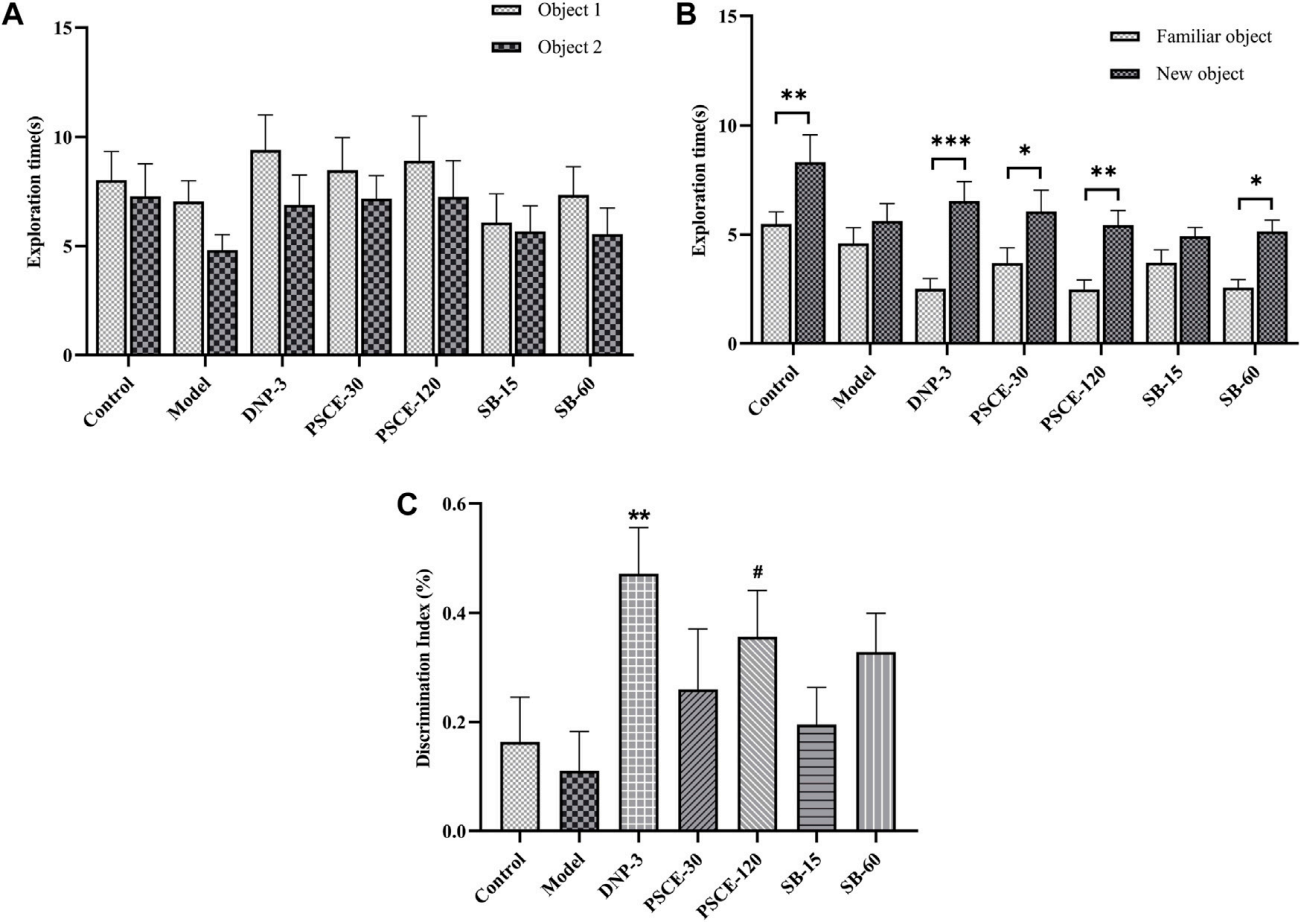
Effects of PSCE and SB on scopolamine-induced mice in NOR. **(A)** the exploration time of two objects in the familiarity period. **(B)** the exploration time of familiar and new objects in the test period. **(C)** the relative discrimination index (DI) in the test period. Experimental data were expressed by Means ± SEM (n = 9–13 per group). Compared with the exploration time of the old object, *p < 0.05, **p < 0.01, ***p < 0.001.

In the familiar stage, there was no significant difference in the exploration time of the two objects among each group, as shown in Figure 7A. In the test stage, the model group showed no significant difference in the time spent exploring familiar and new objects, while the control group showed a significant difference (*p* < 0.01). After drug treatment, mice injected with scopolamine were better at recognizing new objects, PSCE 30 mg∙kg-1, PSCE 120 mg∙kg-1, and SB 60 mg∙kg-1 groups spent significantly longer exploring the new object than the familiar object (*p* < 0.05, *p* < 0.01, *p* < 0.05), as shown in Figure 7B; Compared with control group, the DI of mice in model group decreased, but there was no significant difference. Compared with the model group, the DI of mice in the drug treatment group was increased, among which PSCE 120 mg∙kg-1 groups showed significant differences (*p* < 0.05), as shown in Figure 7C.

#### PSCE and SB decreased the latency and increased the number of crossing the platform in the MWM

**FIGURE 8:**
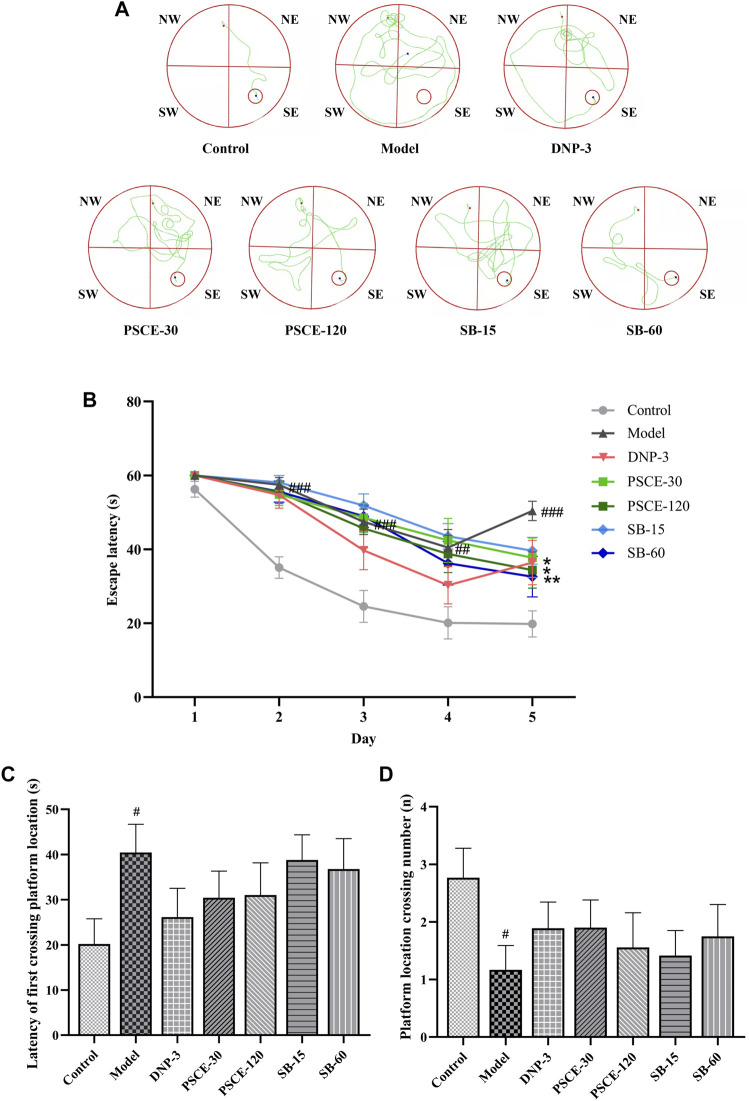
Effects of PSCE and SB on various indexes of scopolamine-induced mice in MWM. **(A)**the swimming track of the acquisition training. **(B)** the escape latency of the acquisition training. **(C)** the escape latency of the probe trial. **(D)** the time of crossing the target platform in the probe trial. Data were expressed by Means ± SEM (n = 9–13 per group), compared with blank group, ^#^ *p* < 0.05; compared with model group, *p < 0.05, **p < 0.01, ***p < 0.001.

As shown in Figure 8B, during the 5-days acquisition training, the escape latency of all groups was shortened with the increase of the experimental days. The track plot for the acquisition training is shown in Figure 8A; The results of repeated measure ANOVA showed that the group factor and time factor had an interaction on the escape latency (*p* = 0.0001), indicating that the effect of group factor changed with time factor. Then the statistical results of the main effect were meaningless, and the group effect on each day was measured by one-way ANOVA to localize which day showed group differences. From the first day of the acquisition training, scopolamine injection significantly prolonged the escape latency in the model group compared with the control group (*p* < 0.01). On the fifth day of the acquisition training, compared with the model group, PSCE 120 mg∙kg-1 and SB 60 mg∙kg-1 groups significantly shortened the escape latency (*p* < 0.05, *p* < 0.01).

As shown in Figures 8C,D, in the probe trial, compared with the control group, the latency of first crossing platform location of the model group was significantly prolonged (*p* < 0.05), and the number of crossing the platform location was significantly decreased (*p* < 0.05). Compared with the model group, the latency of first crossing platform location of the drug treatment group was shortened to different degrees, and the number of crossing the platform location was increased, but no significant difference was shown.

### PSCE and SB regulated the scopolamine-induced cholinergic system in the brain tissue

**FIGURE 9:**
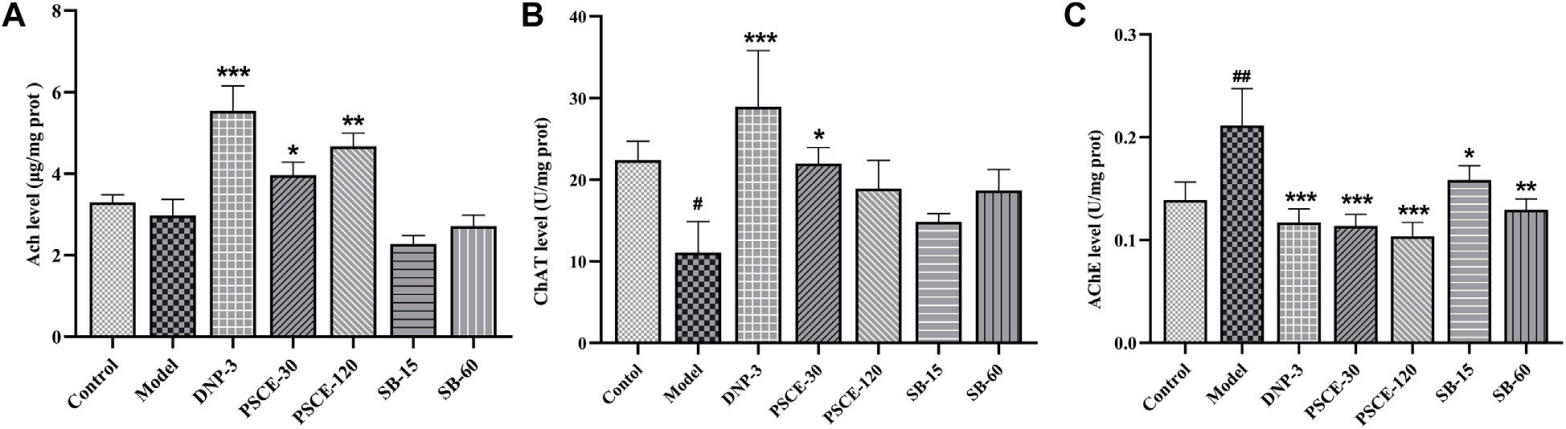
Effects of PSCE and SB on scopolamine-induced cholinergic index. **(A)** Ach activity in cerebral cortex. **(B)** ChAT activity in cerebral cortex. **(C)** AChE activity in cerebral cortex. The experimental data were expressed by Means ± SEM (n = 10 per group). Compared with normal group, ^*#*^ *p* < 0.05, ^##^ *p* < 0.05; Compared with model group, *p < 0.05, **p < 0.01, ***p < 0.001.

As shown in Figure 9, compared with the control group, Ach level and ChAT activity in the brain tissue of the model group decreased significantly (*p* < 0.05). In contrast, the activity of AChE was significantly increased (*p* < 0.01). Compared with the model group, Ach level in brain tissue was significantly increased in PSCE 30 mg∙kg-1 and PSCE 120 mg∙kg-1 groups (*p* < 0.05, *p* < 0.01). ChAT activity was significantly increased in PSCE 30 mg∙kg-1 groups (*p* < 0.05). AChE activity was significantly decreased in PSCE 30 mg∙kg-1, PSCE 120 mg∙kg-1, SB 15 mg∙kg-1, and SB 60 mg∙kg-1 group (*p* < 0.001, *p* < 0.001, *p* < 0.05, *p* < 0.01).

### PSCE and SB decreased scopolamine-induced oxidative stress in the brain tissue

**FIGURE 10:**
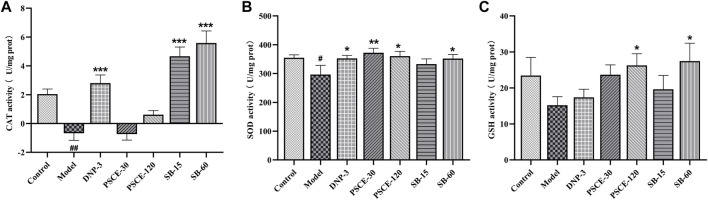
Effects of PSCE and SB on scopolamine-induced oxidative stress state. **(A)** CAT level in brain. **(B)** SOD level in brain. **(C)** GSH level in brain. Experimental values were expressed by mean ± SEM (n = 10 per group). Compared with the control group, ^#^ *p* < 0.05; Compared with model group, *p < 0.05, **p < 0.01, ***p < 0.001.

As shown in Figure 10, compared with the control group, CAT and SOD activities in the brain tissue of the model group were decreased significantly (*p* < 0.001, *p* < 0.05), and GSH levels were reduced, showing an increased degree of free radical damage. Compared with the model group, the oxidative stress damage of the brain tissues was improved in the drug treatment group, among which SB 15 mg ? kg-1 and SB 60 mg ? kg-1 group of mice significantly increased CAT activity in brain tissue (*p* < 0.001); PSCE 30 mg∙kg-1, PSCE 120 mg∙kg-1 and SB 60 mg∙kg-1 groups increased considerably SOD activity (*p* < 0.01, *p* < 0.05, *p* < 0.05), and GSH levels in the brain tissue of mice with PSCE 120 mg∙kg-1 and SB 60 mg∙kg-1 were significantly increased (*p* < 0.05).

### PSCE and SB attenuated scopolamine-induced neuroinflammation in the serum

**FIGURE 11:**
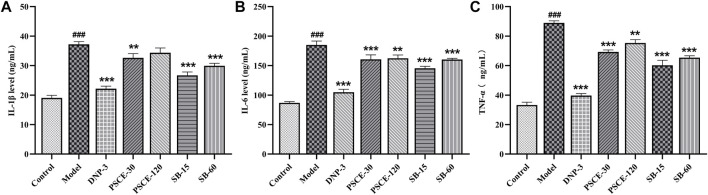
Effects of PSCE and SB on scopolamine-induced neuroinflammation. **(A)** serum IL-6 level. **(B)** serum TNF-α level. **(C)** serum IL-1β level. Compared with control group, ^###^ *p* < 0.05; Compared with model group, **p < 0.01, ***p < 0.001.

As shown in Figure 11, the levels of IL-1β, IL-6, and TNF-α in the serum of the model group were significantly higher than those in the control group (*p* < 0.001). The levels of IL-1β, IL-6, and TNF-α in the serum of the drug treatment group were significantly lower than those in the model group (*p* < 0.01), except for PSCE 120 mg∙kg-1 group.

## Discussion

High-throughput sequencing has certain advantages in studying multi-component, multi-target, and multi-pathway systems of natural products. It can play a predictive role in function and mechanism by combining bioinformatics (Koehn and Carter, 2005). Therefore, this study used high-throughput screening technology to find genes with significant changes in PSCE and 10 stilbenes-treated at the transcriptional level. Then, the biological functions, signaling pathways, and diseases enriched by DEGs were found through GO, KEGG, and disease enrichment analysis. The signaling pathways related to AD were further analyzed to find the main active components that play a role in improving cognitive impairment. In this study, multiple DEGs were detected by comparing RNA-seq in the drug-treated and untreated groups, alterations in gene expression induced by PSCE, and 10 stilbenes in HepG2 cells. GO enrichment analysis showed that DEGs were widely involved in various biological functions. Disease enrichment analysis showed that DEGs on PSCE and 10 stilbenes were significantly enriched in cancer, AD, type 2 diabetes, etc. Other reports showed consistently that PSCE could significantly inhibit the proliferation of oral squamous carcinoma cells (Ren and Qing, 2020), improve cognitive impairment in scopolamine-induced mice (Liu et al., 2020), and lower starch mediated PBG levels in diabetic mice (Liu et al., 2021). Stilbenes also showed the same results in cancer (Kim et al., 2002; Almosnid et al., 2016; Tian et al., 2020), diabetes (Liu et al., 2021; Wang et al., 2022), and arthritis (He et al., 2019) as in this study. KEGG pathway enrichment analysis showed that DEGs of PSCE were significantly enriched in p53, HIF-1, MAPK, PI3K/Akt signaling pathway, etc. Studies have shown that the p53 signaling pathway could maintain REDOX homeostasis, regulate inflammation, control synaptic function, and reduce Aβ production (Iyalomhe et al., 2017); Activation of PI3K-Akt signaling pathway can protect against Aβ-induced neurotoxicity and help delay the progression of AD (Long et al., 2021); MAPK signaling pathway can increase Tau phosphorylation and amyloidogenesis, and neuroinflammation, activate microglia and astrocytic cell, causing neuron damage and synaptic dysfunction and accelerate the process of AD (Chan et al., 2019; He et al., 2020); HIF-1 signaling pathway is the downstream signal of PI3K-Akt signaling pathway and MAPK signaling pathway, which can enhance the survival ability of cells under hypoxia, inhibit neuronal apoptosis, improve cerebral blood flow, and improve oxidative stress and inflammatory injury state (Iyalomhe et al., 2017). These indicated that p53, HIF-1, MAPK, and PI3K-Akt signaling pathways were the critical signaling pathways, and PSCE might play a role in improving cognitive impairment by affecting apoptosis, neuroinflammation, oxidative stress homeostasis, and synaptic function.

KEGG pathways were enriched in compounds **1**, **2**, **5**, **6**, **7**, **8**, **9**, and **10** rather than compounds **3**, **4**. Disease enrichment analysis showed that DEGs of compounds **2**, **4**, **5**, **7**, **8** and **10** were significantly enriched in neurodegenerative disorder. According to previous studies, compounds **1**, **2**, **3**, **6**, **9**, and **10** were more abundant in PSCE than the other stilbenes (Liu et al., 2020). Combined with the above results, it was speculated that compounds **2** (suffruticosol B) and **10** (*trans*-gnetin H) might be the foremost effective components of PSCE. It would be the critical component in the following study on the anti-AD activity of a single component.

It was worth noting that HIF-1 signaling pathway was the pathway with the highest frequency of enrichment, and was also enriched in PSCE, SB and GH, so it was speculated that HIF-1 signaling pathway was the critical pathway for SB and GH to play a medicinal role. The molecular docking results showed that the binding energies of SB and GH were lower than -5 kcal·mol-1, indicating high affinity between SB, GH and the target proteins in HIF-1 signaling pathway. HIF-1 signaling pathway can improve inflammation and oxidative stress injury and protect neuronal cells. Moreover, target proteins enriched in this pathway also showed AD-related activity. IL6 and ERBB2 can participate in the inflammatory response during AD (Papassotiropoulos et al., 2001; Chaudhury et al., 2003); EIF4EBP1 can regulate cell proliferation (Pause et al., 1994); ENO2 can have neurotrophic and neuroprotective effects (Takano et al., 2016); the levels of neurotrophic factors EDN1 and HIF1A in the cerebral cortex are upregulated, while VEGFA levels were down-regulated during AD, leading to dysregulation of vascular homeostasis and angiogenesis (Hibbs et al., 2021; Tsartsalis et al., 2021). HK2 down-regulation is related to the disorder of aerobic glycolysis in the AD brain, which can cause dysregulation of energy metabolism (Li et al., 2021). These results suggested that SB and GH might play a role in AD prevention by acting on HIF-1 signaling pathway. Combined with previous studies SB had stronger β-secretion *in vitro* and cholinesterase inhibitory activity (Choi et al., 2011; Liu et al., 2020; Tian et al., 2020). Therefore, our study took SB as an example to explore the effects of PSCE and SB on improving cognitive impairment.

Scopolamine is a competitive blocker of M-type acetylcholine receptors, which can lead to cholinergic dysfunction (Lei and Xiaoli, 2020), oxidative damage (Baek et al., 2020), mitochondrial dysfunction (Yamagami et al., 2021), apoptosis (Fronza et al., 2020), neuroinflammation (Olayinka et al., 2022), suppression of trophic factors (Yamagami et al., 2021), leading to severe impairment of learning and memory (Tang, 2019). In the present study, mice injected intraperitoneally with scopolamine showed prominent impairments in learning and memory skills as well as in the cholinergic system, oxidative stress and neuroinflammation of the brain tissues, which proved that the scopolamine-induced mouse model was a good choice for AD study. PSCE and SB in the present study played an important role in attenuating dementia and biochemical parameters induced in scopolamine-induced mice.

NOR was used to evaluate experimental animals’ non-spatial and short-term learning and memory abilities (Taoro-Gonzalez et al., 2019). In the present study, it was revealed that scopolamine reduced mice’s preference for new object as indicated by DI and the time mice spent exploring objects. After PSCE and SB treatment, the mice’ preference for new objects increased, which suggested that PSCE and SB could improve the short-term and non-spatial discernible memory of scopolamine-induced mice. MWM is the most classic behavioral research method in neuroscience, which is used to evaluate the learning and memory ability of the animals for spatial position and direction sense (Meng et al., 2019). The latency of scopolamine-induced mice was significantly prolonged, and the number of platform location crossings was significantly reduced. PSCE and SB could shorten not only latency but also increase the number of platform crossings. Results suggested that PSCE and SB could improve the learning and memory ability of spatial position and direction sense in scopolamine-induced mice. The present study further confirmed the findings by Liu (Liu et al., 2020) on the enhancements of learning and memory abilities in mice with PSCE.

To further explore the improvement of PSCE and SB on scopolamine-induced learning and memory impairment in mice, we detected biochemical indicators related to cholinergic system, oxidative stress and neuroinflammation. The cholinergic injury hypothesis is one of the classical hypotheses on the pathogenesis of AD. The leading cause of memory loss is the loss of cholinergic neurons in the brain of AD patients, which is manifested by decreased Ach level, decreased ChAT activity, and increased AChE activity (Hampel et al., 2018). The present study showed that scopolamine injection could decrease Ach level and ChAT activity and increase AChE activity, which was consistent with the results of other studies (Lv et al., 2021). PSCE and SB could reverse the abnormal changes in the cholinergic system in mice. PSCE and SB were more selective on Ach and AChE than ChAT. The study further confirmed the findings by Liu(Liu et al., 2020) on PSCE and SB inhibiting AChE activity *in vitro*. Then, the disorder of oxidative stress in the brain neurons is significant in the pathogenesis of AD (Joseph et al., 2020), and dysfunction related to oxidative levels has also been reported in AD patients (Mahdi et al., 2021). CAT and SOD are necessary antioxidant enzymes in the brain, and GSH is an essential antioxidant substrate. In present study, scopolamine could reduce the activities of CAT and SOD, and GSH levels in brain tissues, which was consistent with other results (Mushtaq et al., 2018; Mostafa et al., 2021). PSCE and SB could improve oxidation indexes in brain tissues, which further confirmed the antioxidant effect of PSCE and SB *in vitro* (Chunnian et al., 2016; Yang et al., 2022). Furthermore, neuroinflammatory injury is also one of the pathological features of AD. In the brain tissues of AD patients and animal models, a large number of pro-inflammatory factors are released, resulting in tissue damage and further reducing the clearance of Aβ, thereby aggravating Aβ aggregation (Longjian et al., 2020). In present study, scopolamine could increase the levels of TNF-α, IL-1β, and IL-6, which was the same as that of Yang (Yang et al., 2021) and Mostafa (Mostafa et al., 2021). PSCE and SB could reverse neuroinflammatory damage in brain tissues. It was noteworthy that SB could reduce IL-6 levels as evidenced that the previous results of high-throughput sequencing and molecular docking. These results suggested that anti-oxidation might be the critical mechanism of PSCE and SB in improving scopolamine-induced cognitive dysfunction.

Compared with previous studies, the enhancement effect of PSCE at different doses on the cognitive function of mice showed similarities and differences. Compared mice treated with PSCE 150 mg∙kg-1 in the study of Liu(Liu et al., 2020) with mice treated with PSCE 120 mg∙kg-1 in present study, the results of the behavioral test and biochemical indicators were similar, which verified the reliability of the results. The 30 mg∙kg-1 group showed only an improvement trend in behavioral tests, and some indicators showed significance. It suggested that the improvement effect of 30 mg∙kg-1 group was not strong, which needed further exploration in the later stage. It was worth noting that compared with the 600 mg∙kg-1 group, the 150 mg∙kg-1 group showed more significant performance in the inhibitory avoidance test, inflammation index, cholinergic index and partial oxidation index. It was inferred that PSCE could have beneficial effects even at low doses, whereas 600 mg∙kg-1 might not be necessary for cognitive behavior.

## Conclusion

PSCE might exert pharmacodynamic activity through multiple components, targets, and pathways. SB and GH might be the main active components of PSCE and play a pharmacodynamic role through the HIF-1 signaling pathway. PSCE and SB could improve the cholinergic system, inhibit oxidative stress and improve neuroinflammation to reverse scopolamine-induced cognitive dysfunction in mice. The study indicated that PSCE and SB might be potential anti-AD drug candidates. This study provided the basis for stilbenes in the treatment of AD, which avoided the waste of traditional Chinese medicine resources and was conducive to the sustainable development of traditional Chinese medicine resources.

## Data Availability

The data presented in the study are deposited in the SRA repository, accession number SRR20324398.
