# Supplementary material for: Integrating network pharmacology analysis and pharmacodynamic evaluation for exploring the active components and molecular mechanism of moutan seed coat extract to improve cognitive impairment
Source: Front Pharmacol. 2022 Aug 12;13:952876. doi: 10.3389/fphar.2022.952876 (PMC9411852; doi:10.3389/fphar.2022.952876)

Supplementary Material

**
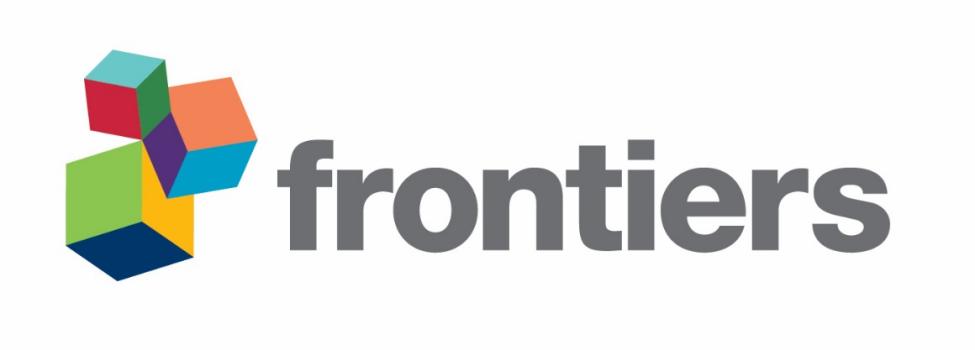
**

**Supplementary Figure 1.** The top 10 significant terms of GO functional enrichment analysis of DEGs on 10 stilbenes. Abscissa: -log(*p*-value), ordinate: GO terms, including biological process (orange), molecular function (green), cellular component (blue). The length of the column indicated the significant -log(*p*-value) of this GO term. The larger the -log(*p*-value) was, the more significant the enrichment was. **(A)** suffruticosol A; **(B)** suffruticosol B; **(C)** suffruticosol C; **(D)** *trans*-resveratrol; **(E)** *cis-ε-*viniferin; **(F)** *trans-ε*-viniferin; **(G)** *cis*-suffruticosol D; **(H)** *cis*-gnetin H; **(I)** *trans*-suffruticosol D; **(J)** *trans*-gnetin H.


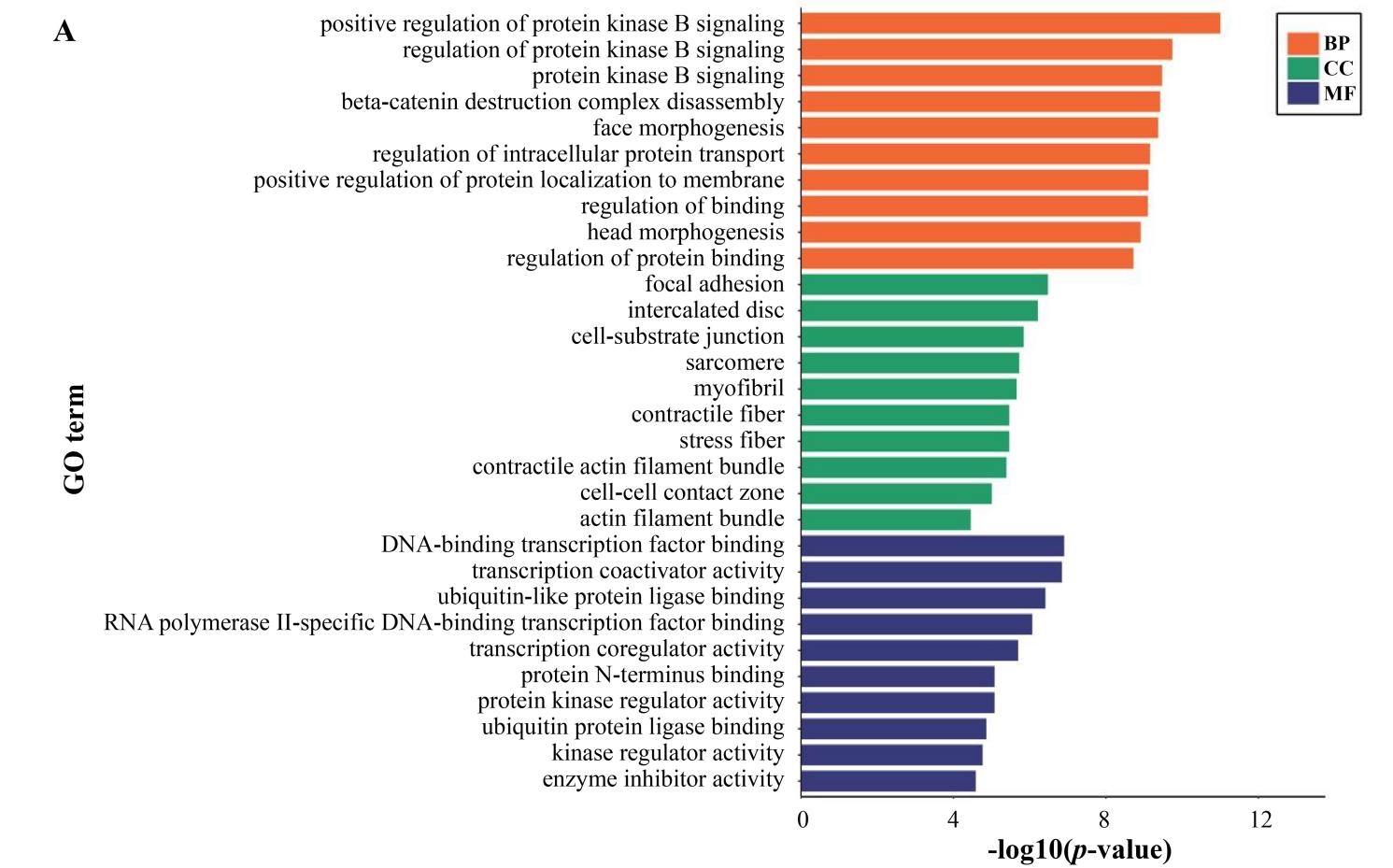


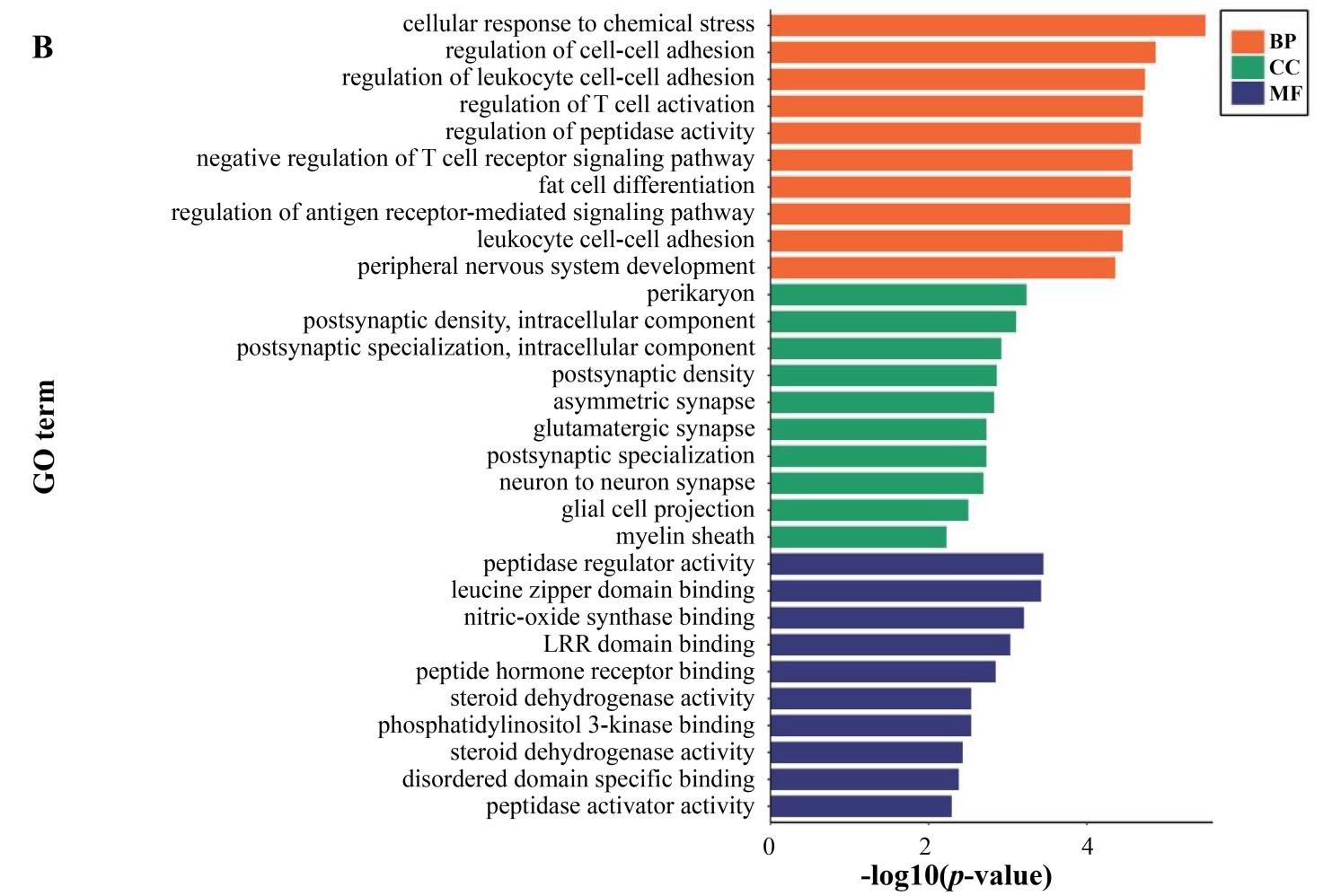


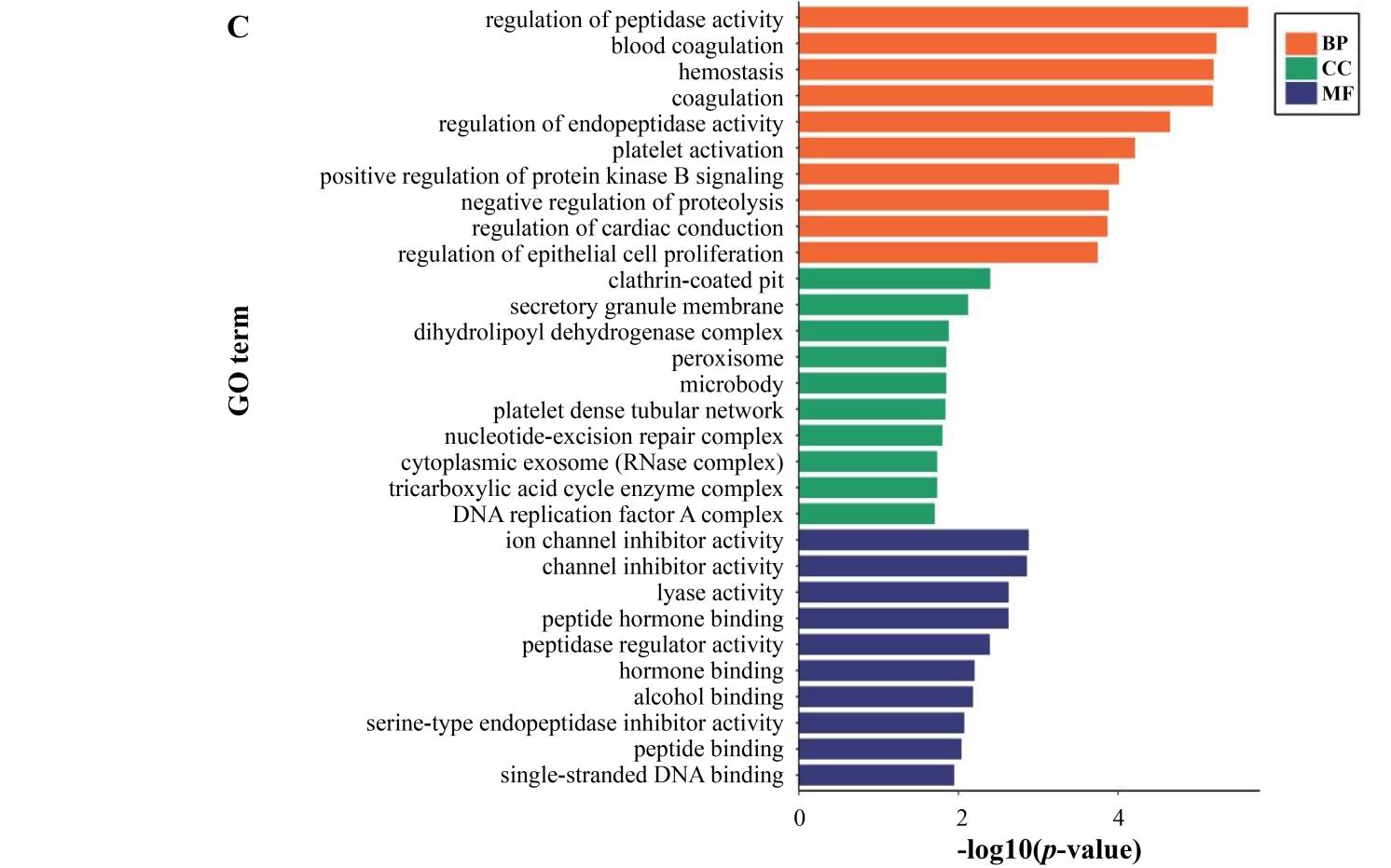


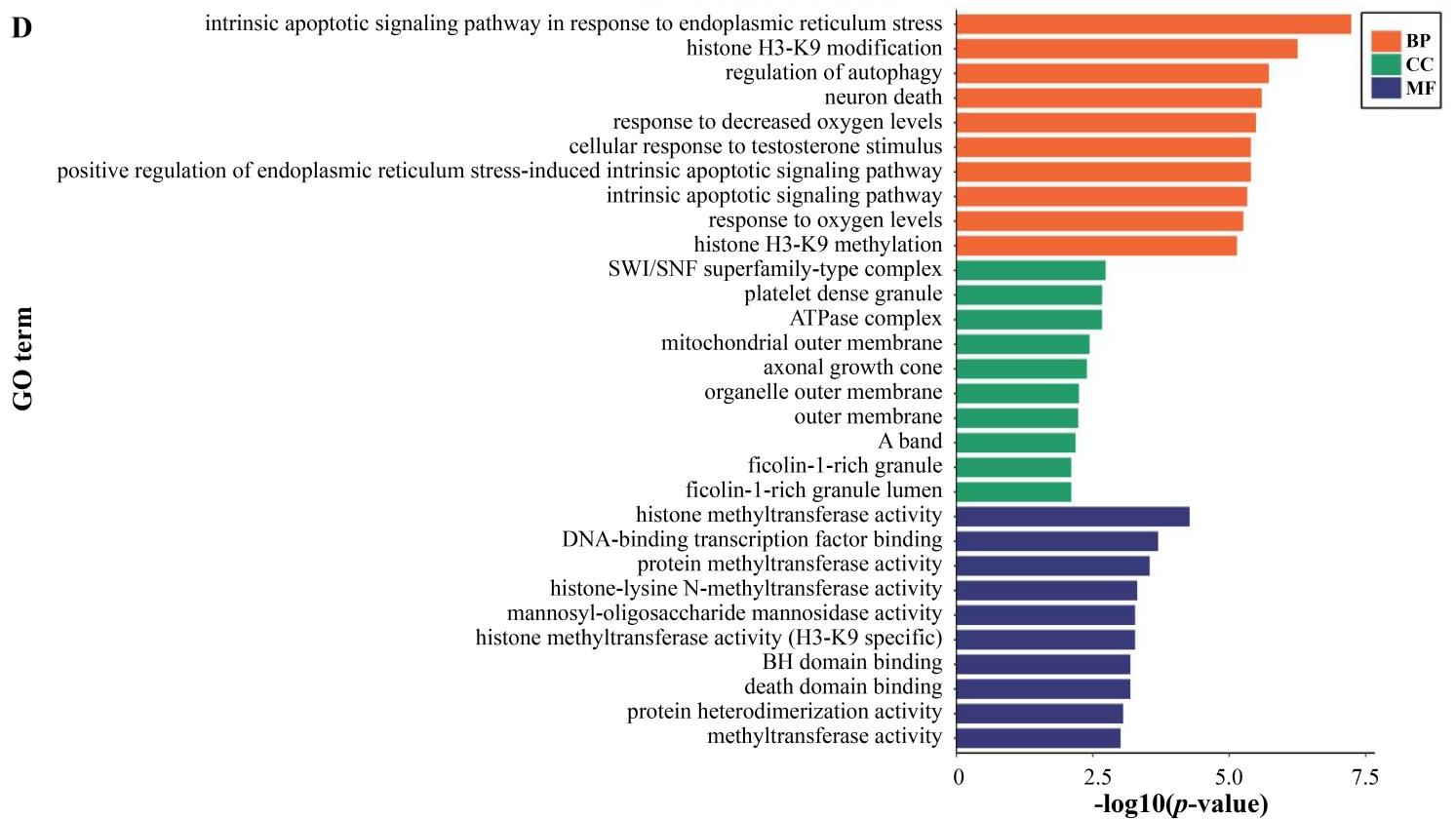


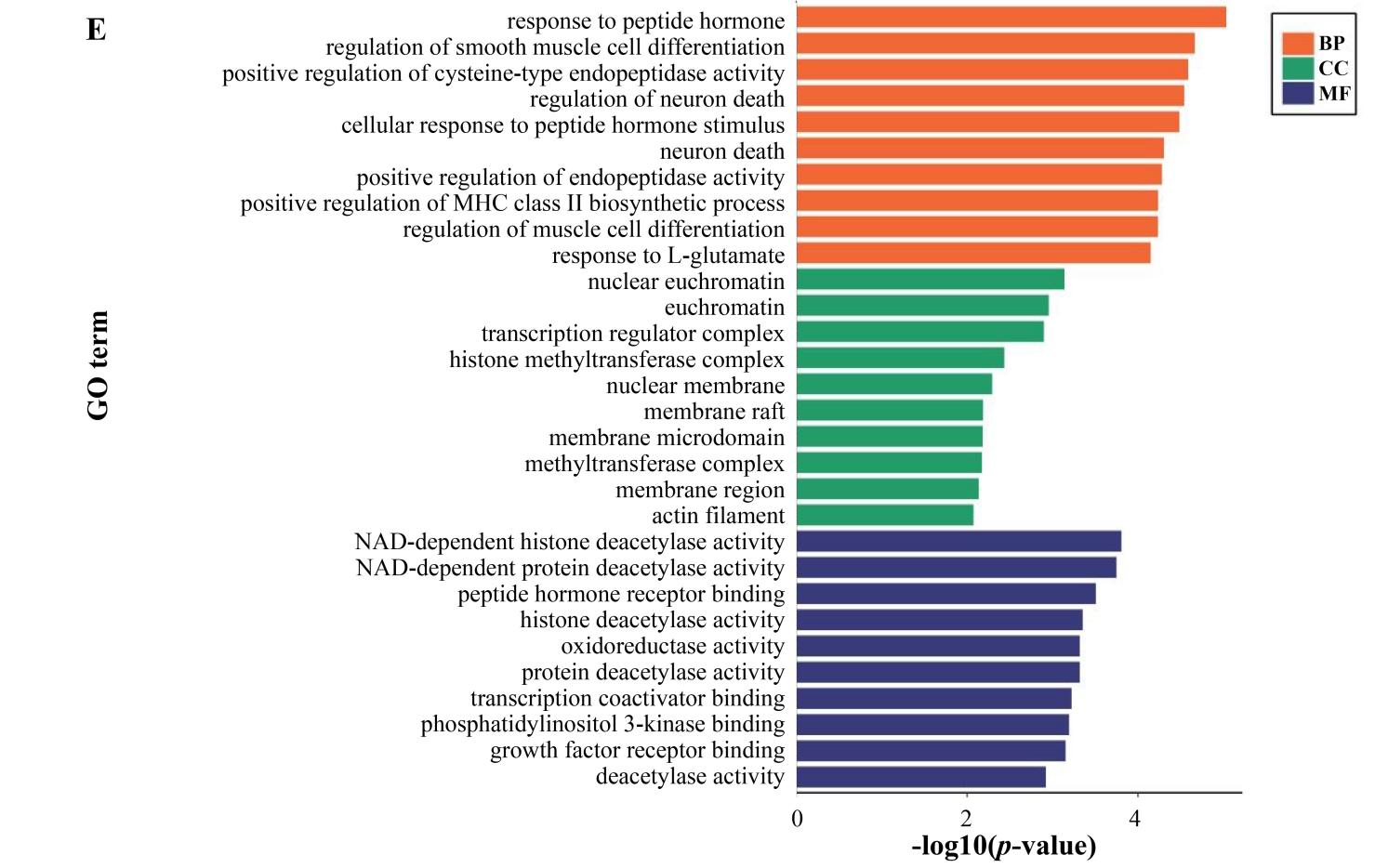


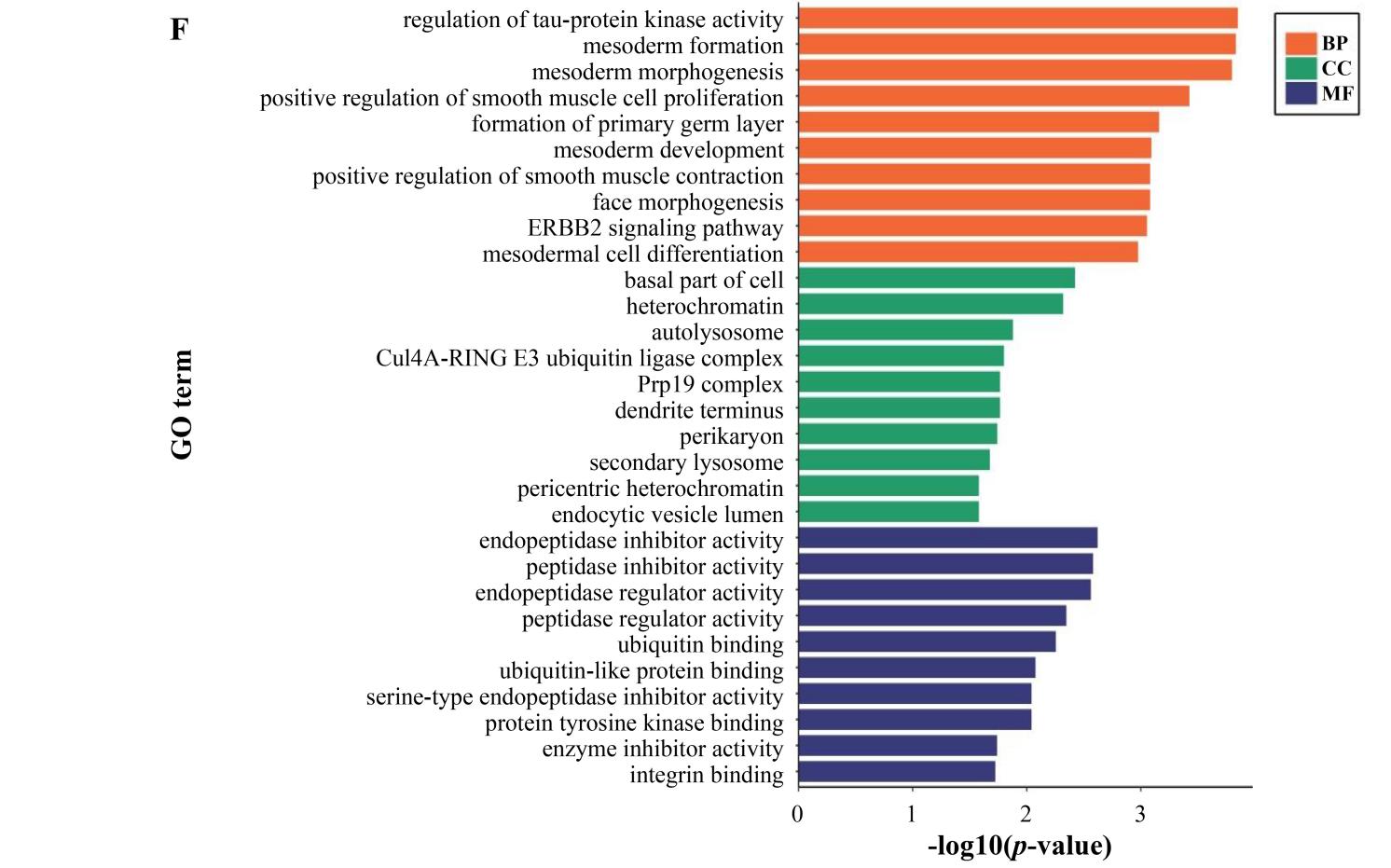


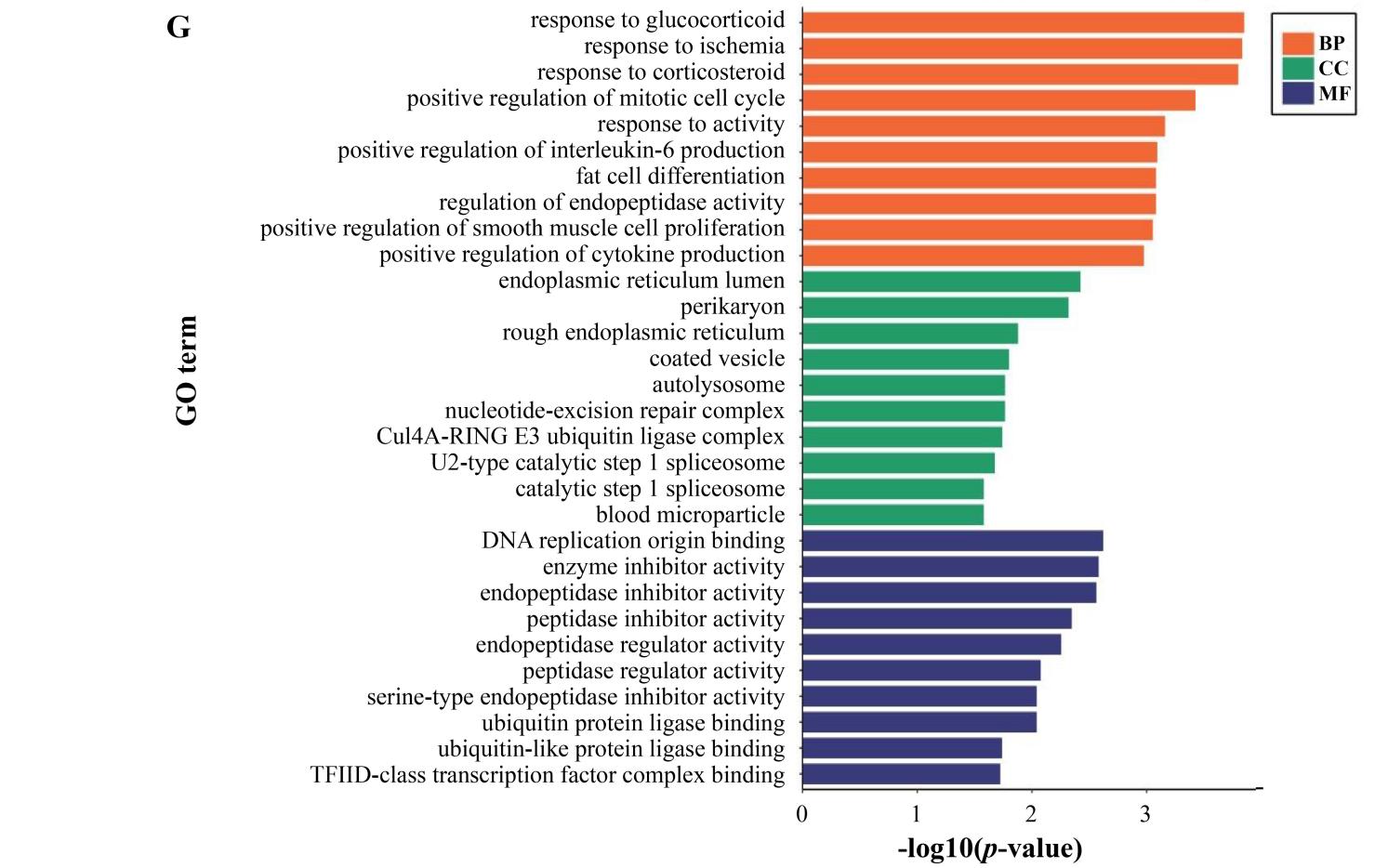


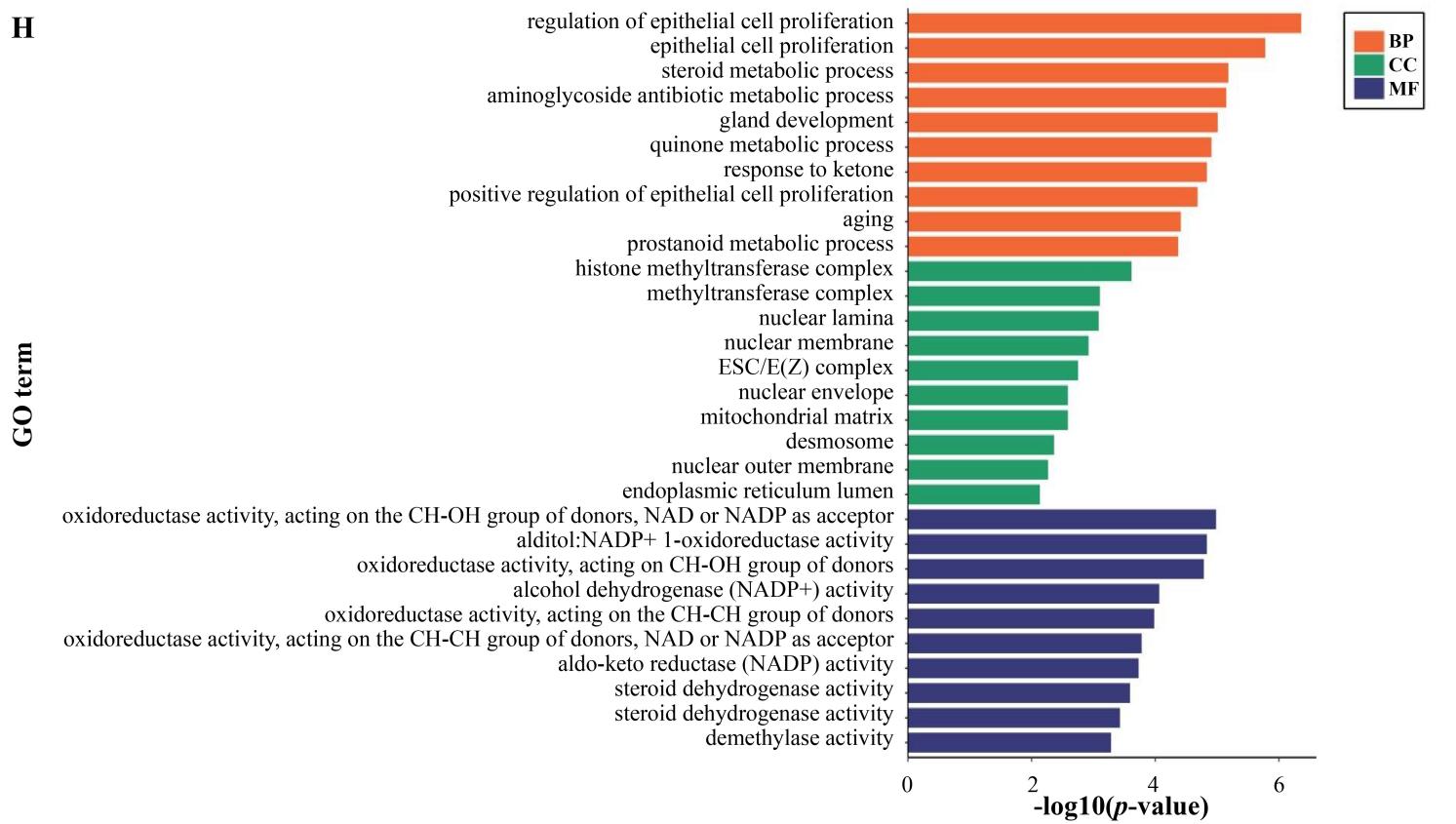


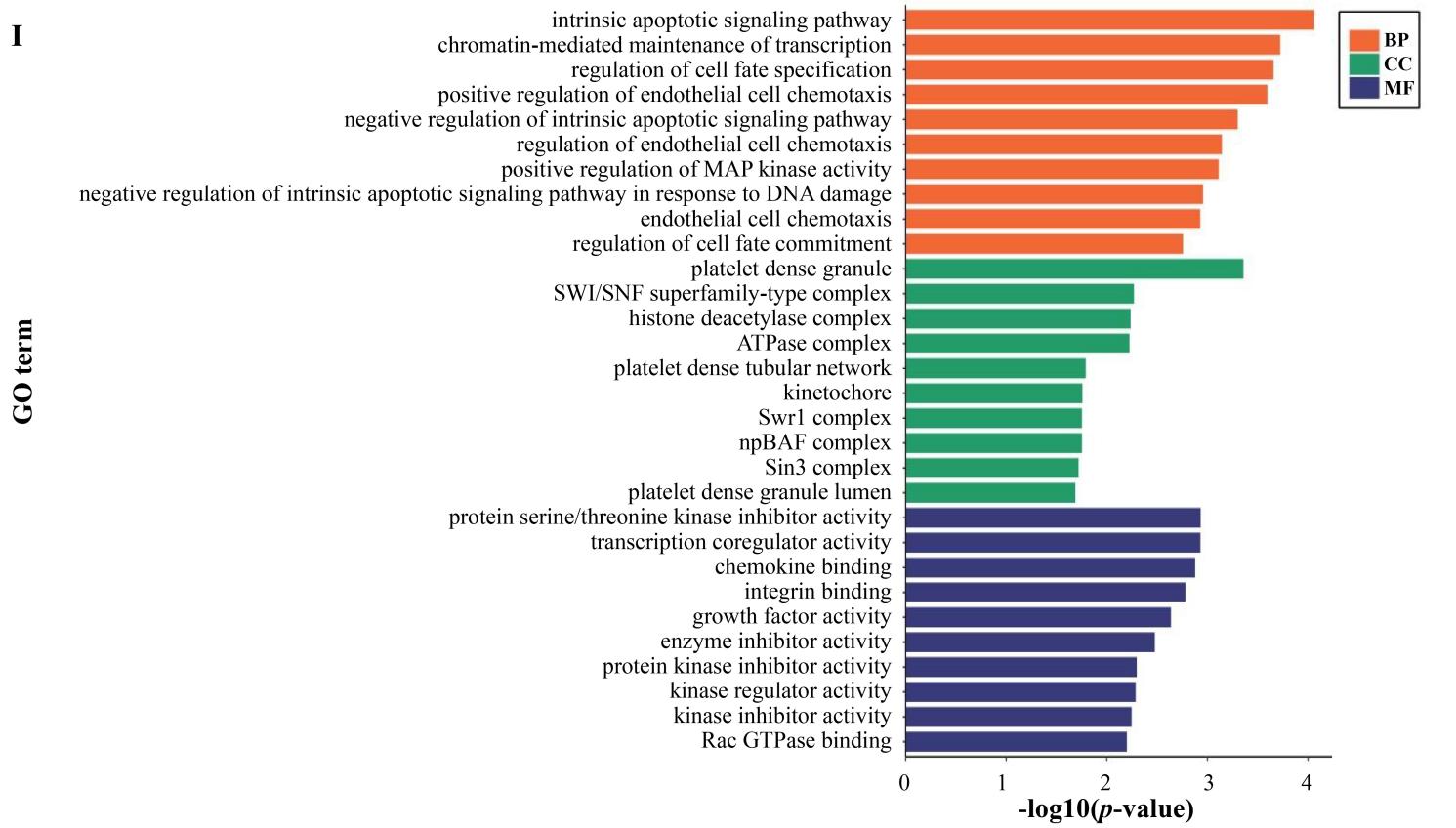


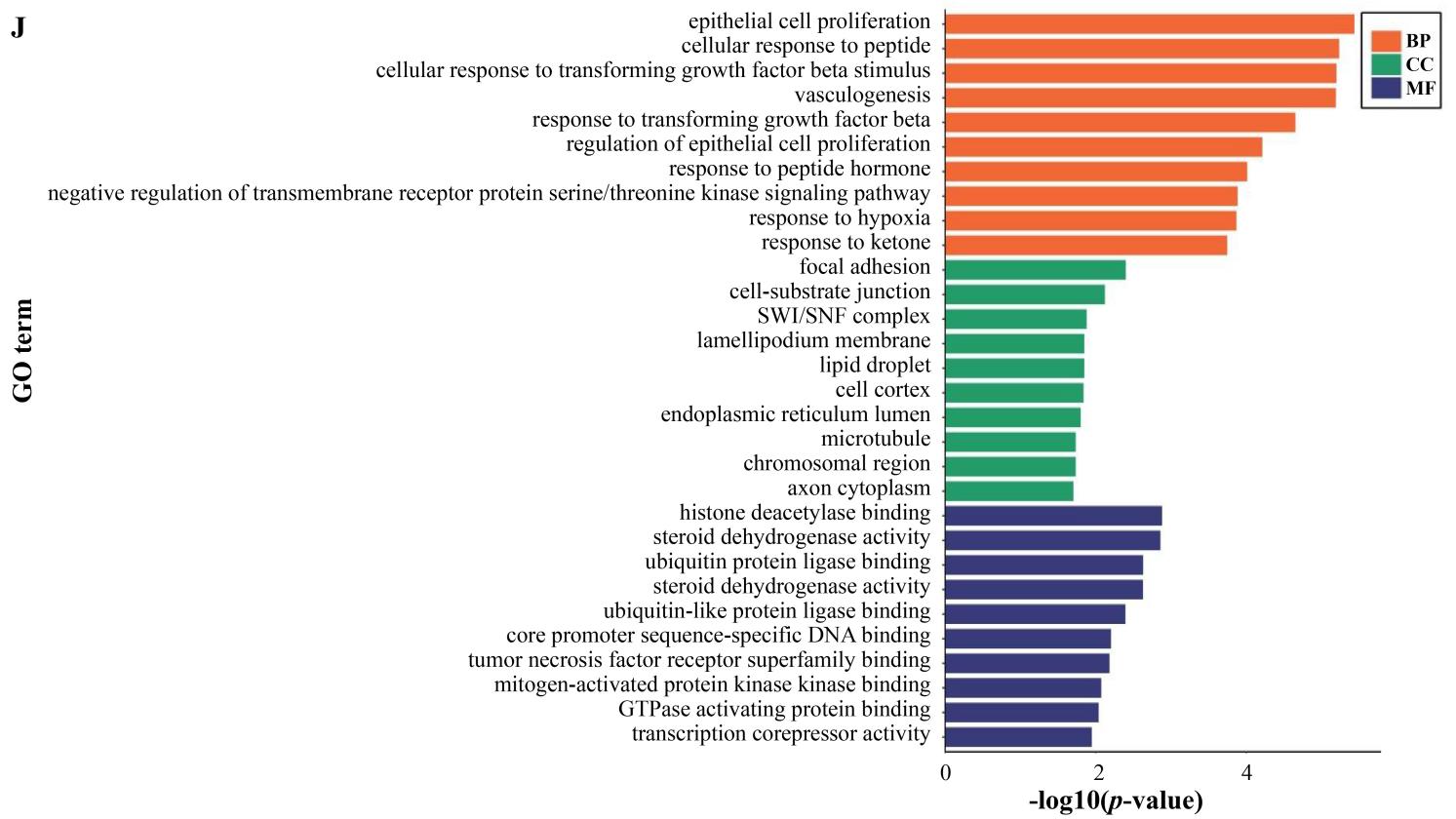


**Supplementary Figure 2.** The top 20 significant terms of KEGG pathway enrichment analysis of DEGs on 10 stilbenes. Abscissa: -log(p-value), ordinate: KEGG pathway. The size of the dot indicated the number of DEGs enriched in the pathway. The color indicates the significant -log(p-value) of this pathway. The larger the -log(p-value) was, the more significant the enrichment was.**(A)** suffruticosol A; **(B)** suffruticosol B; **(C)** suffruticosol C; **(D)** *trans*-resveratrol; **(E)** *cis-ε*-viniferin; **(F)** *trans-ε-*viniferin; **(G)** *cis*-suffruticosol D; **(H)** *cis-*gnetin H; **(I)** *trans*-suffruticosol D; **(J)** *trans*-gnetin H.


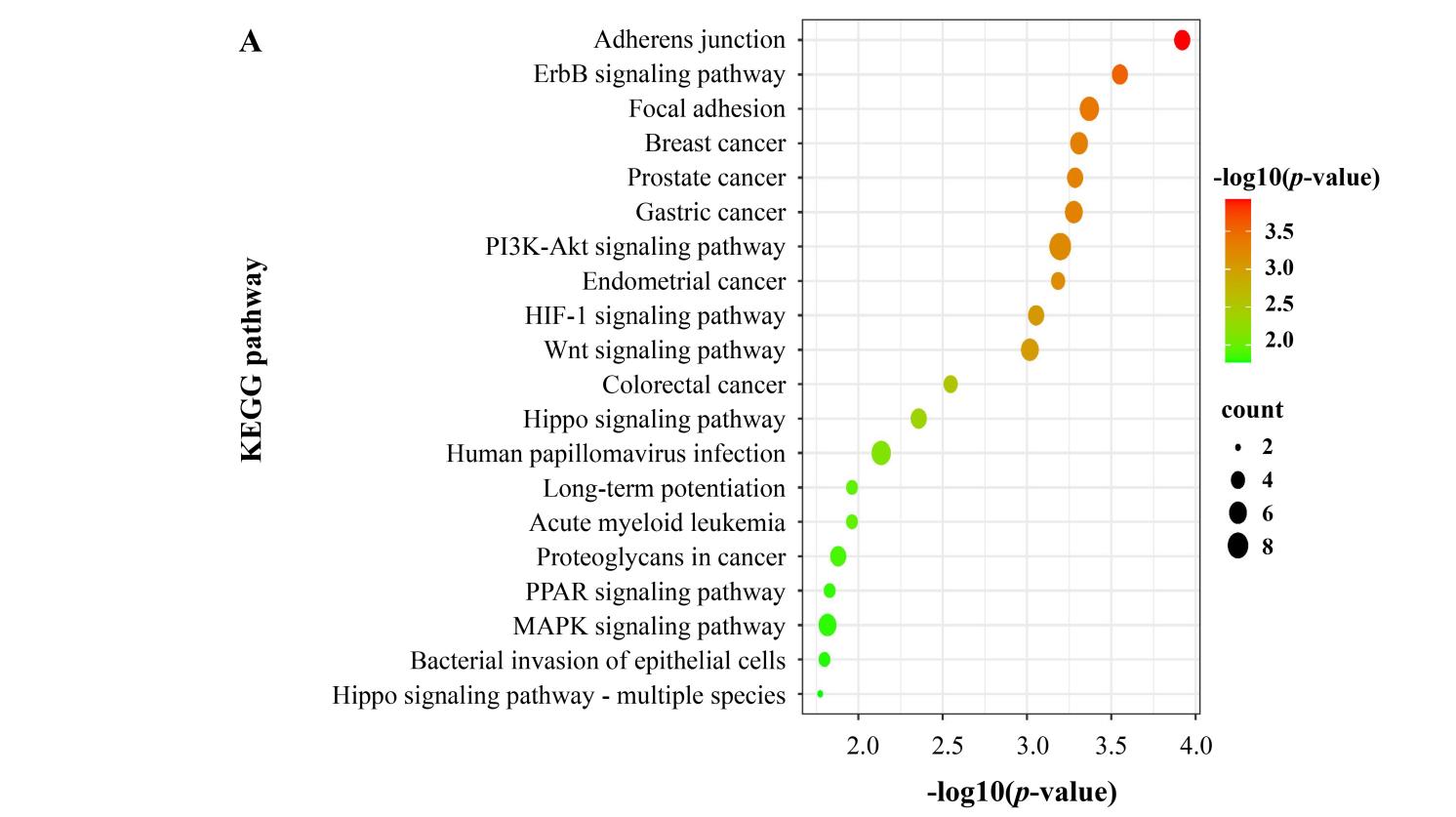


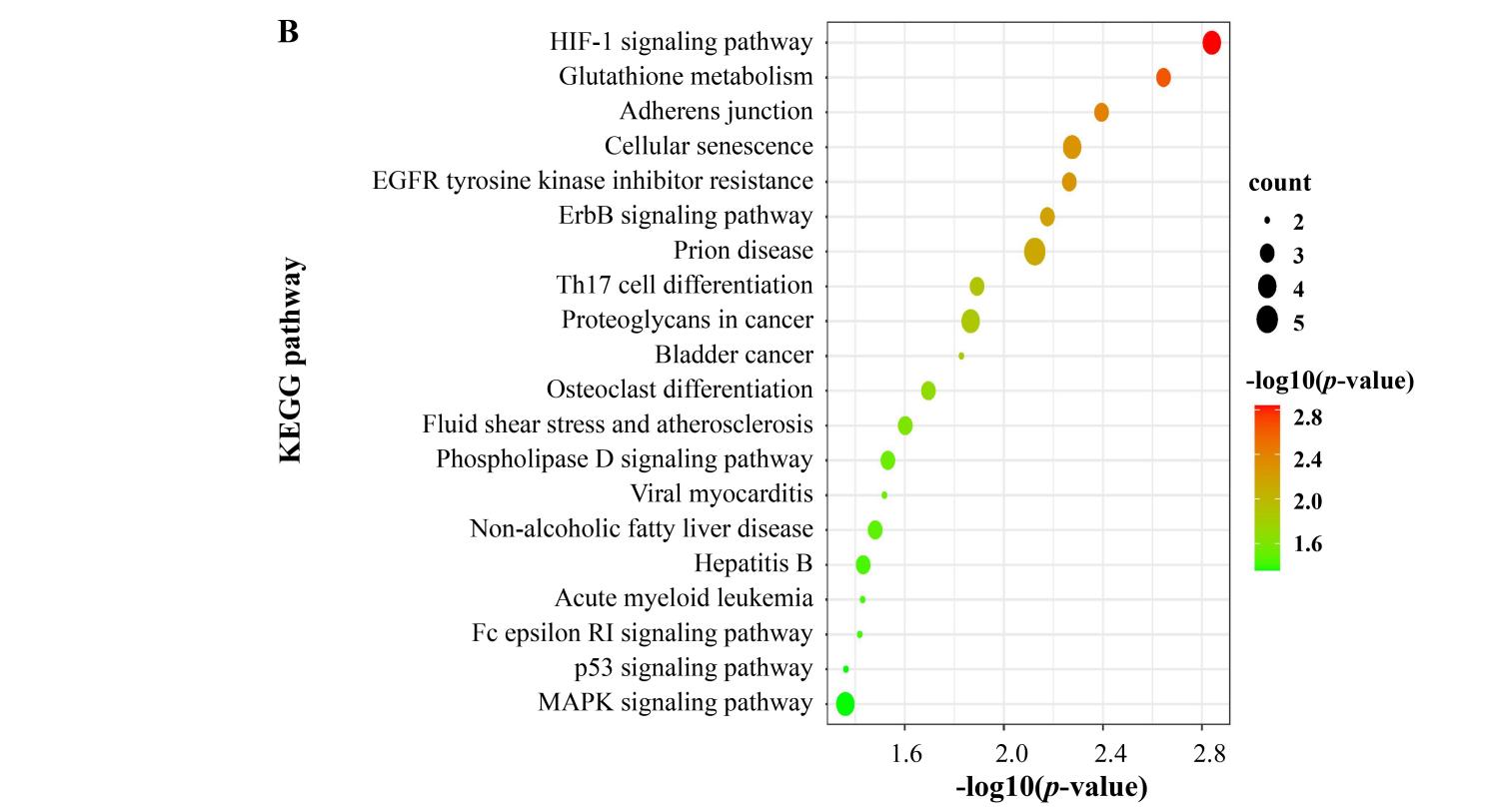


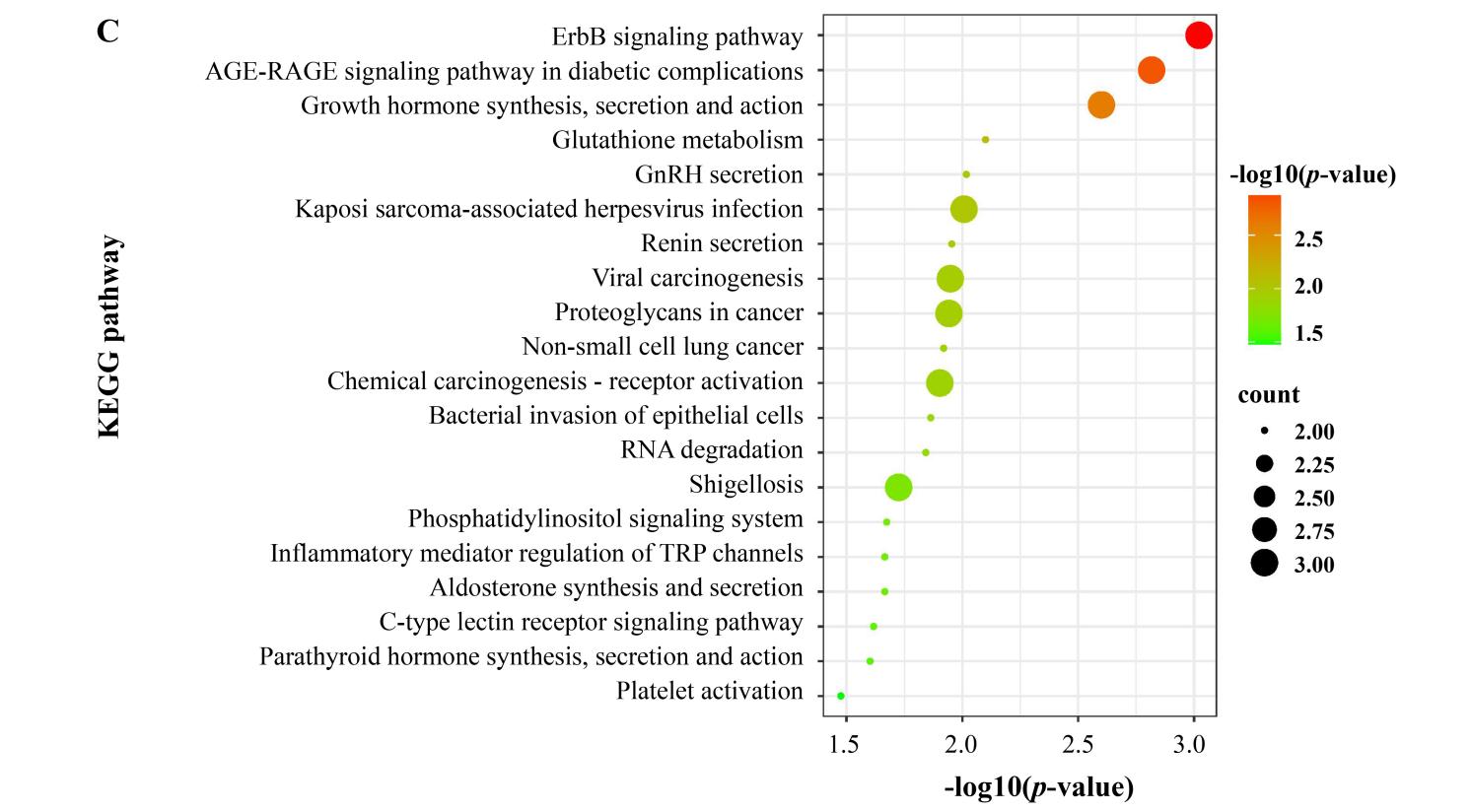


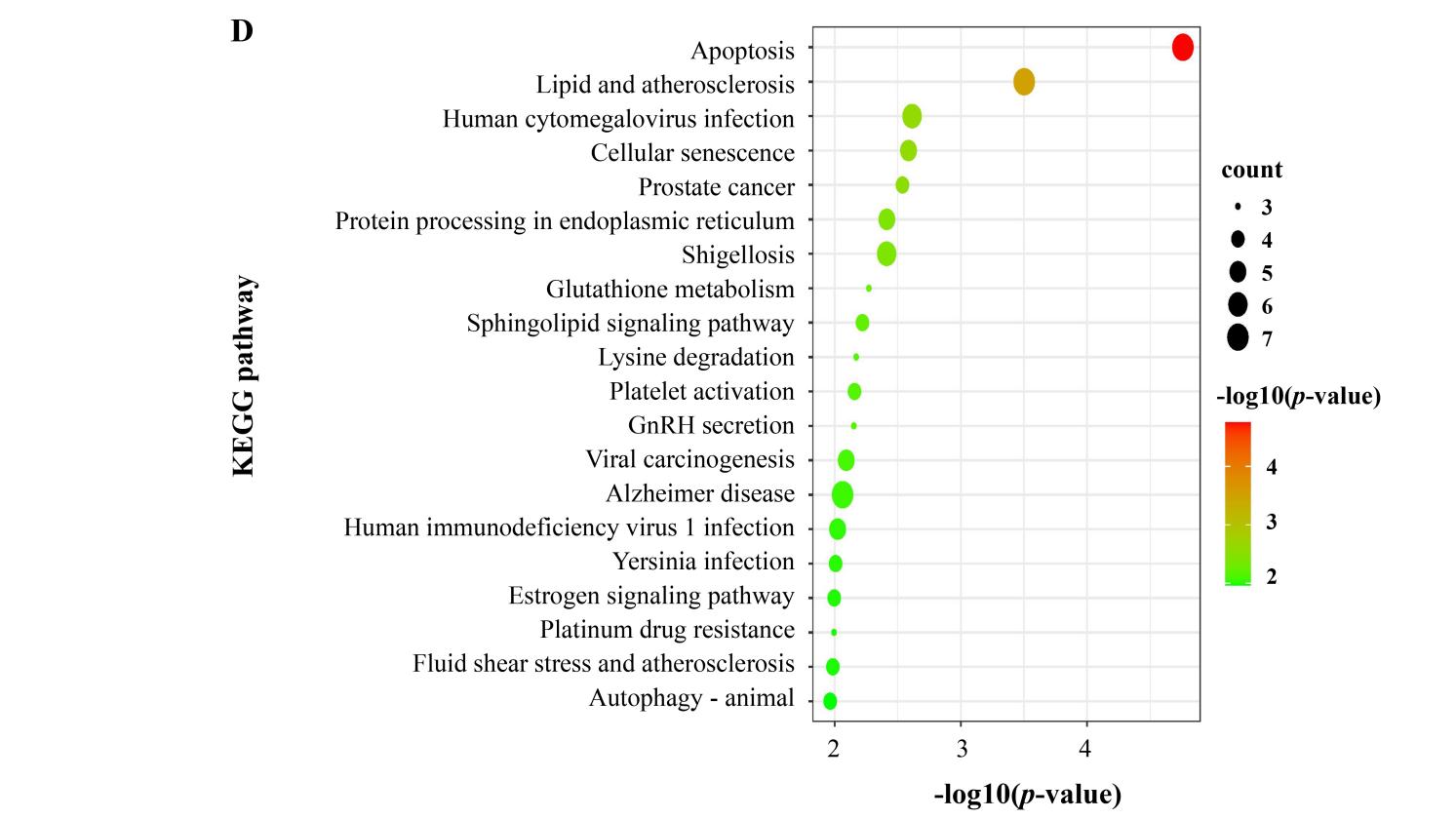


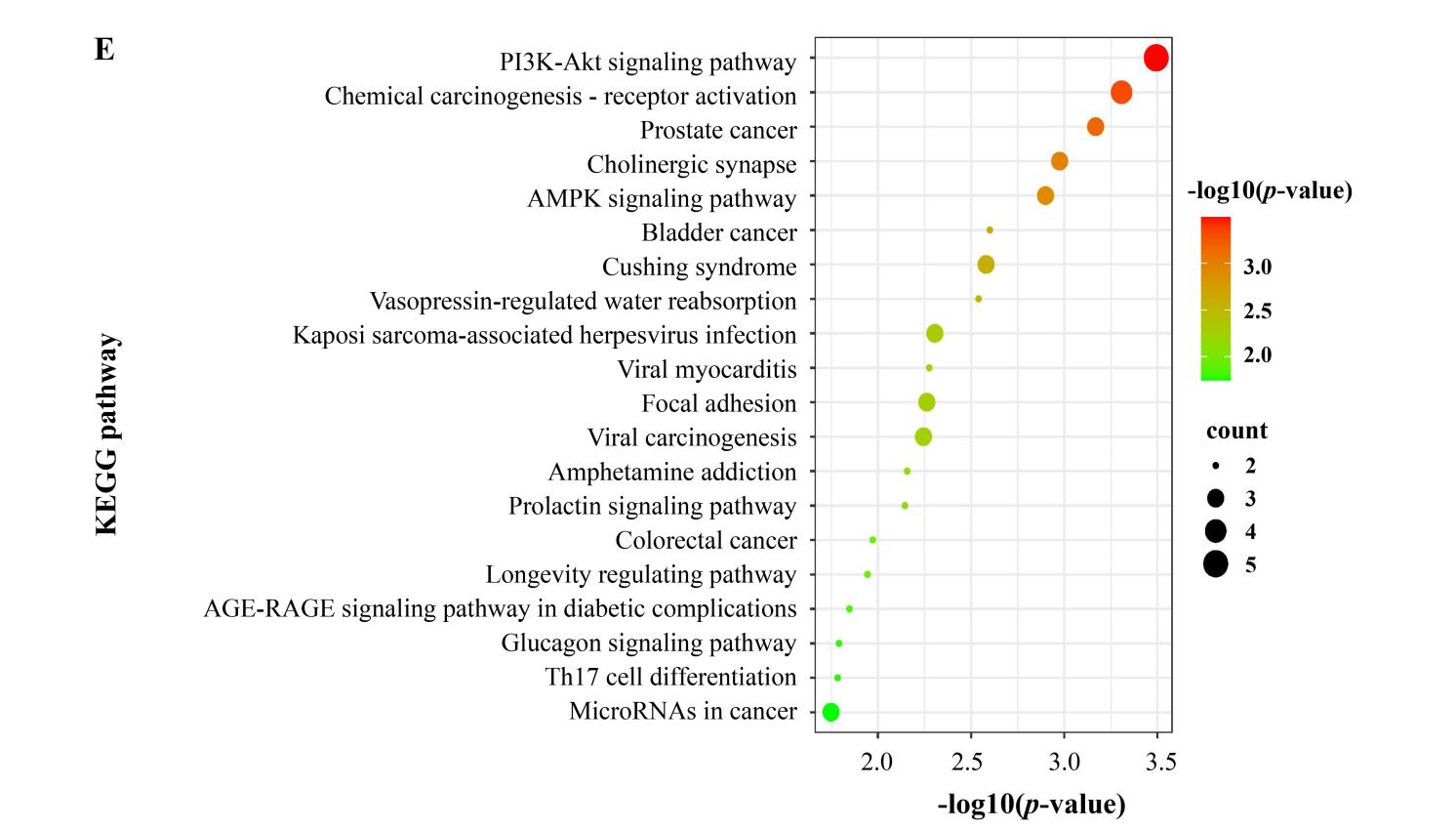


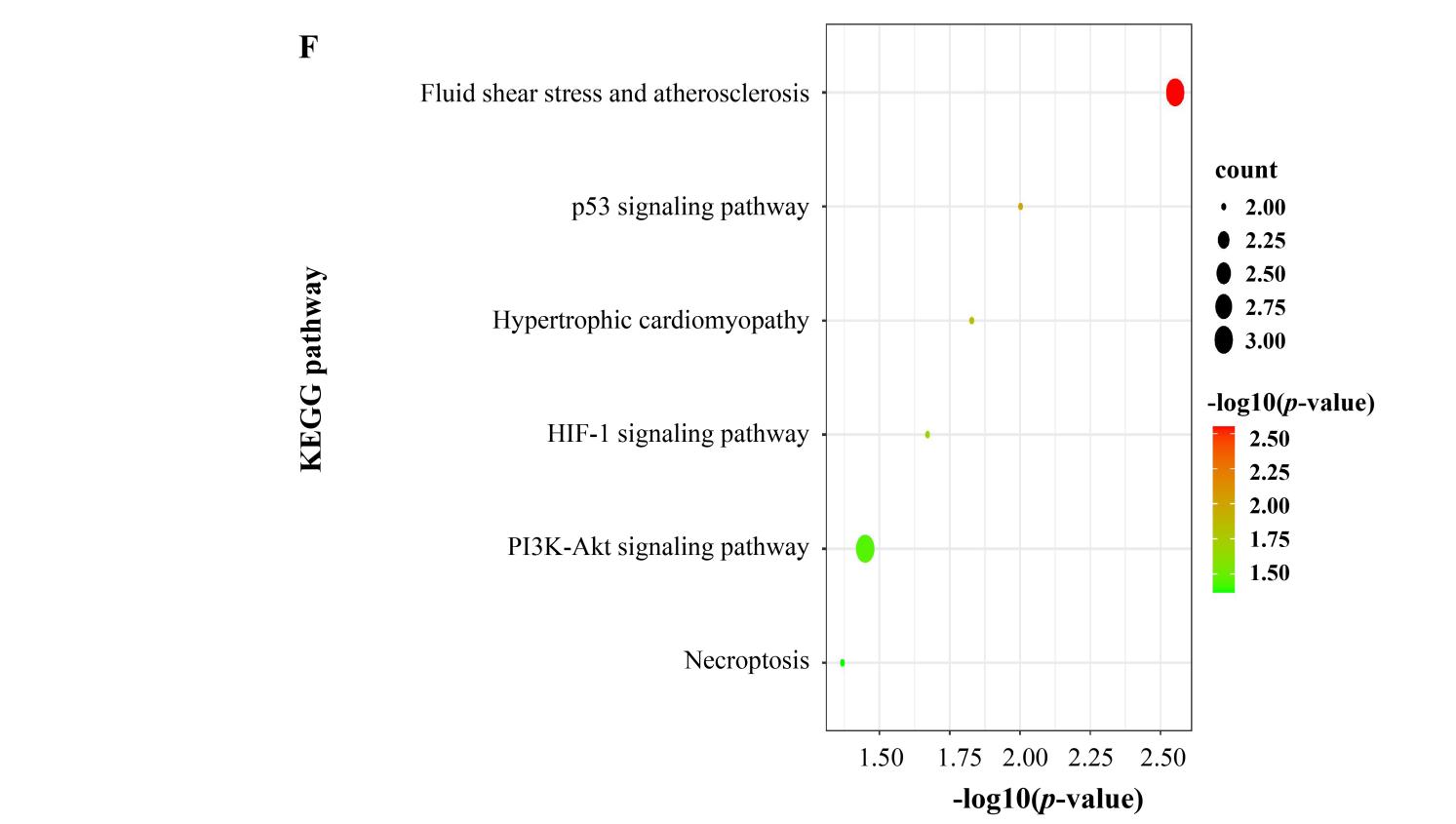


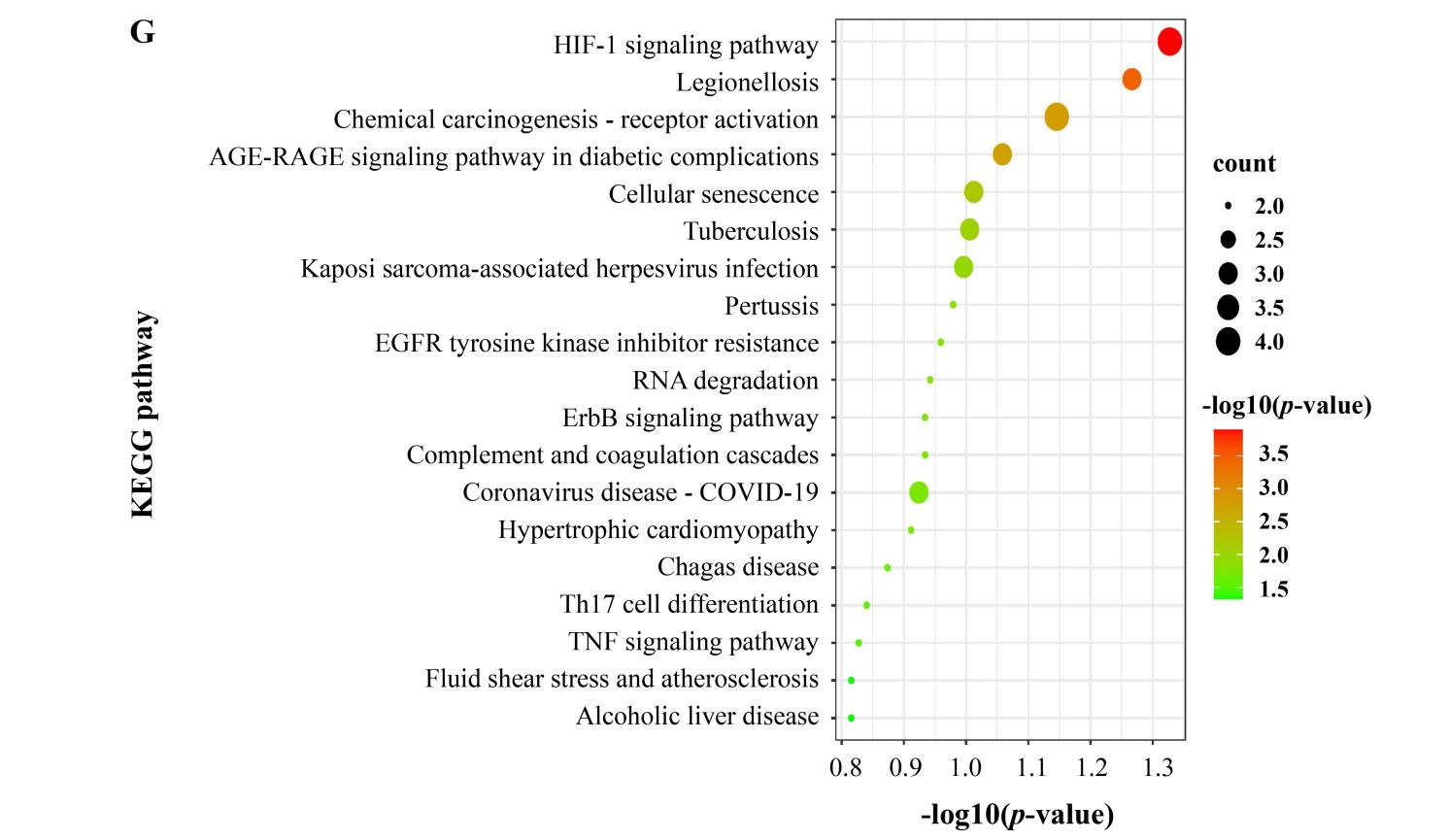


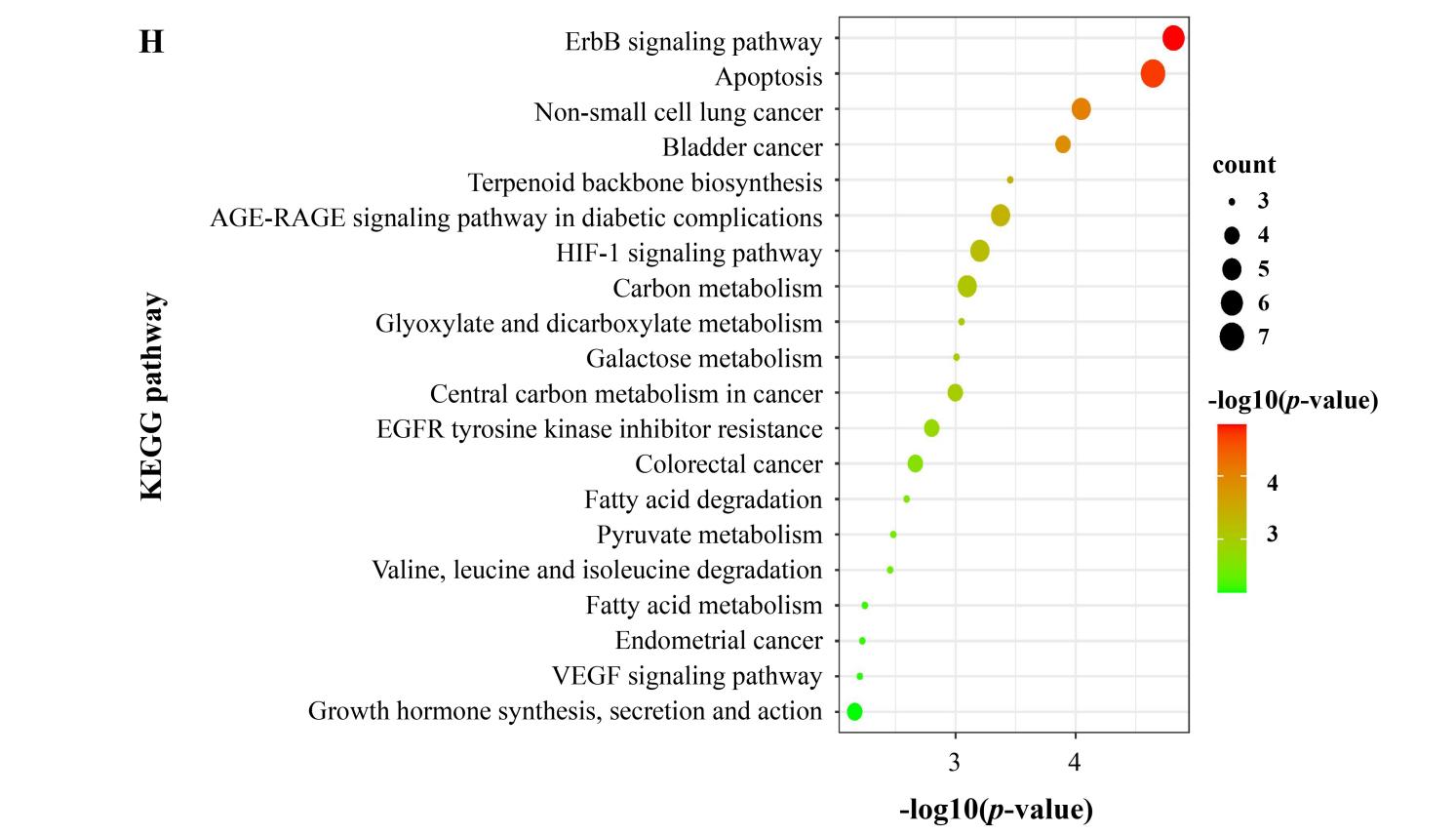


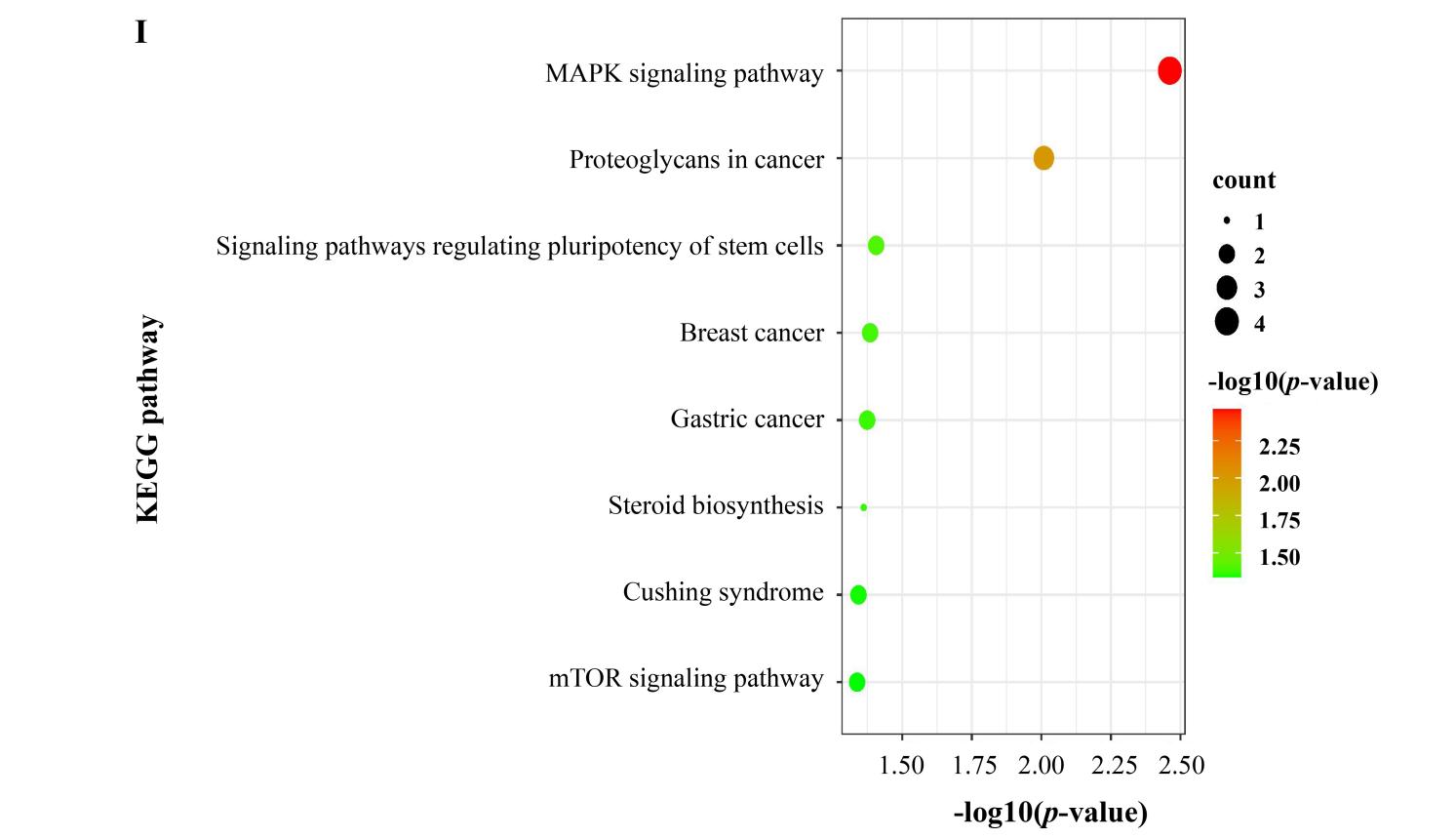


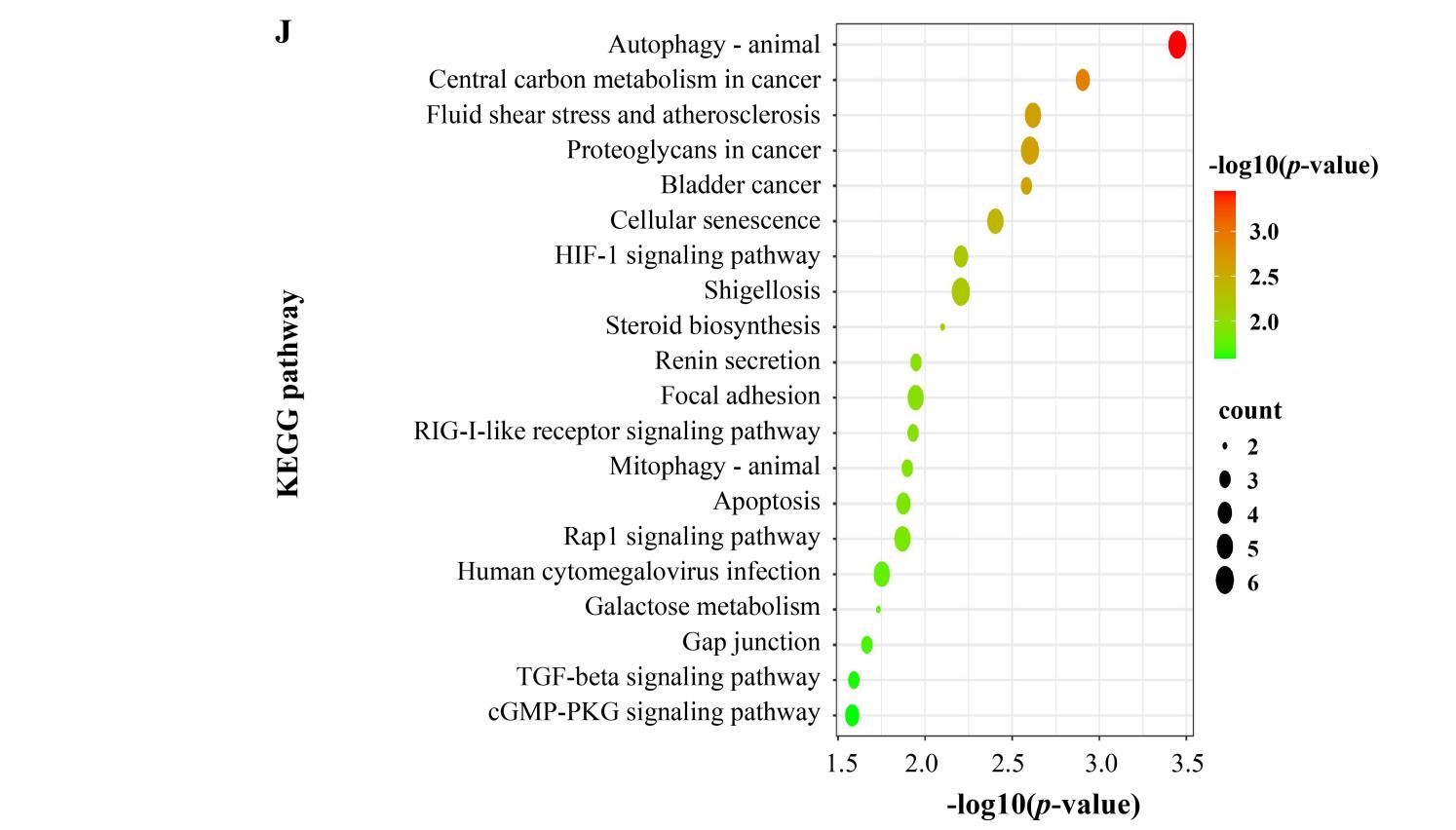


**Supplementary Figure 3.** The top 20 disease enrichment analysis of DEGs on 10 stilbenes. Abscissa: -log(*p*-value), ordinate: disease terms. The length of the column indicated the significant -log(*p*-value) of this disease term. The larger the -log(*p*-value) was, the more significant the enrichment was. **(A)** suffruticosol A; **(B)** suffruticosol B; **(C)** suffruticosol C; **(D)** *trans*-resveratrol; **(E)** *cis-ε-*viniferin; **(F)** *trans-ε*-viniferin; **(G)** *cis*-suffruticosol D; **(H)** *cis*-gnetin H; **(I)** *trans*-suffruticosol D; **(J)** *trans*-gnetin H.


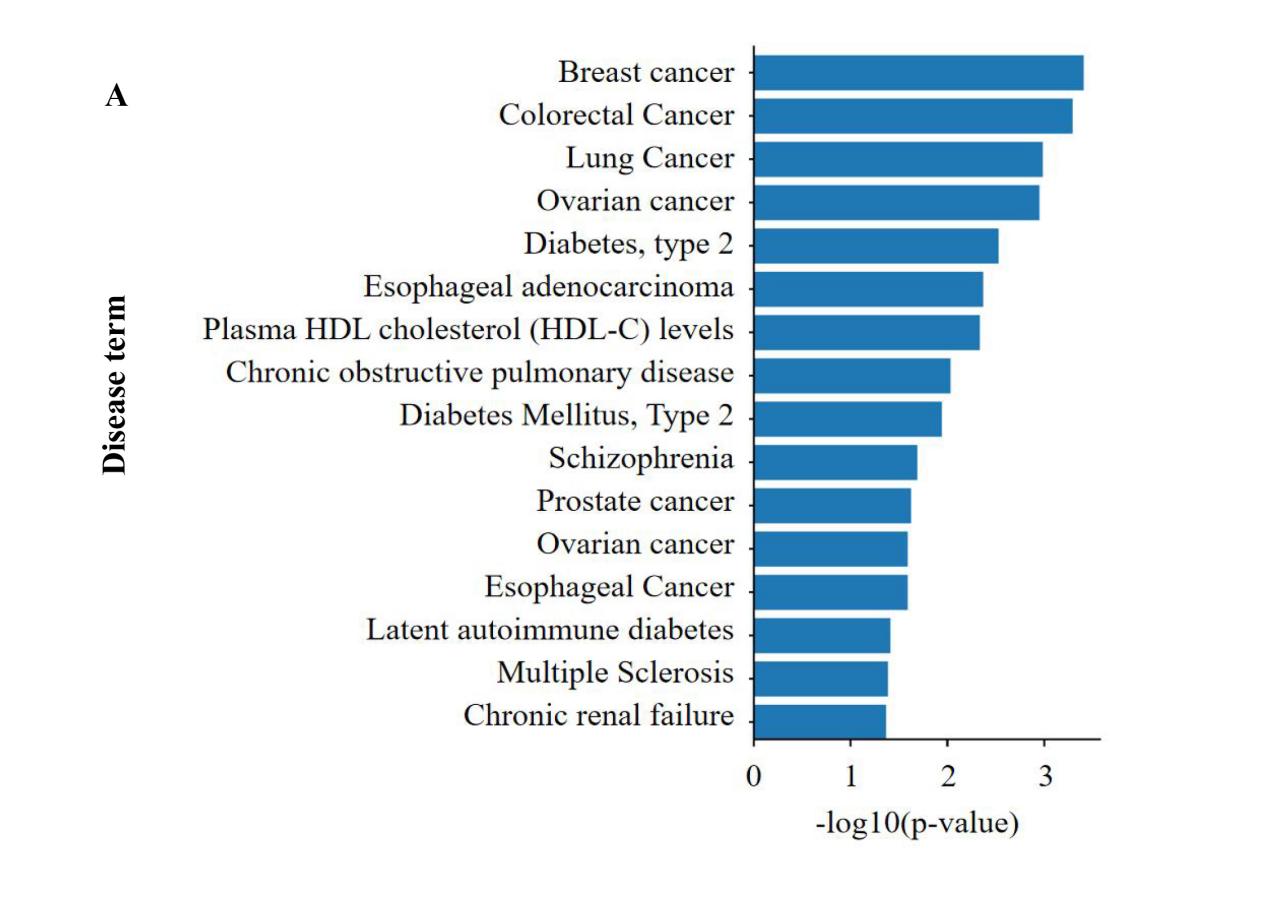

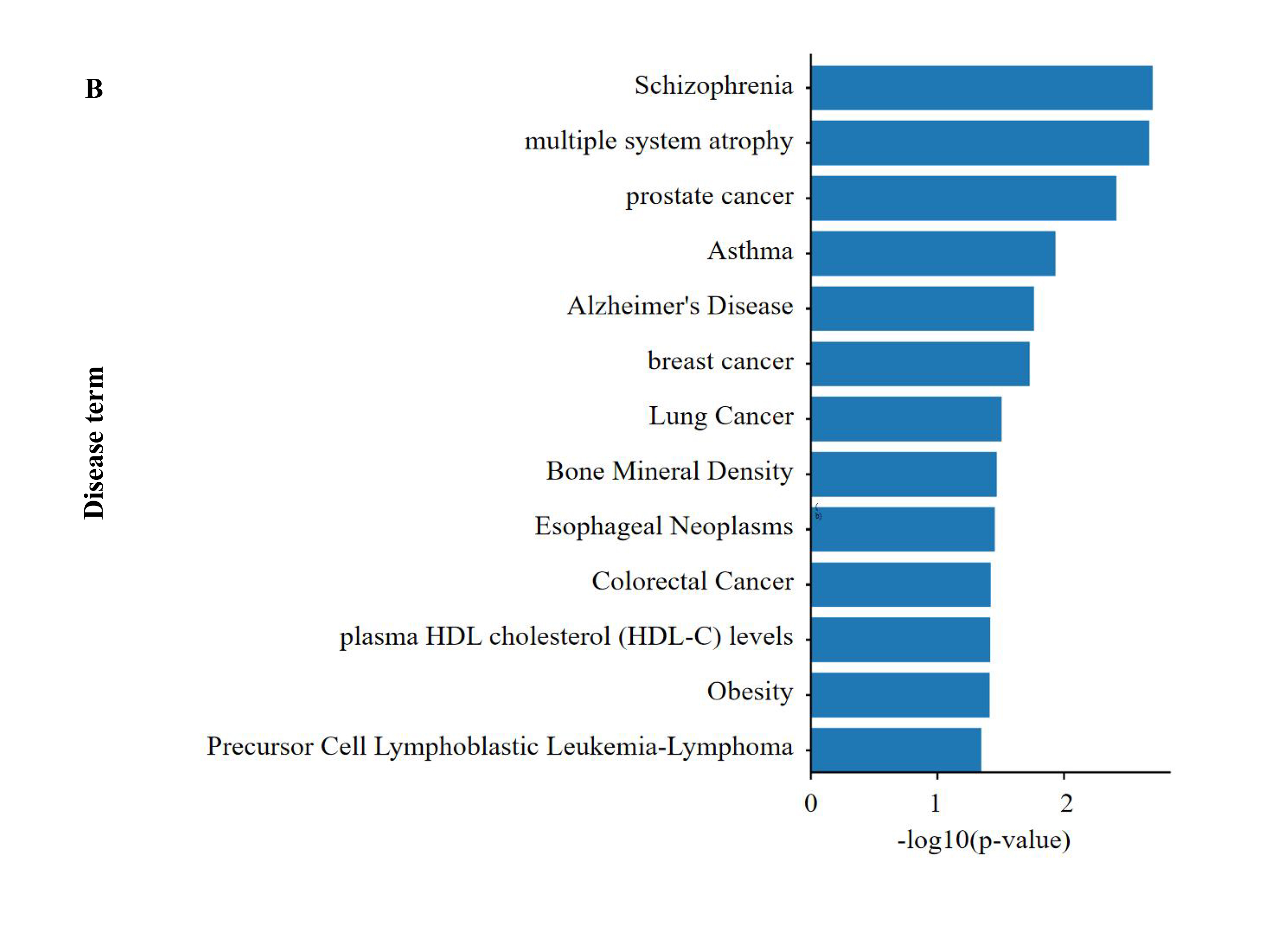


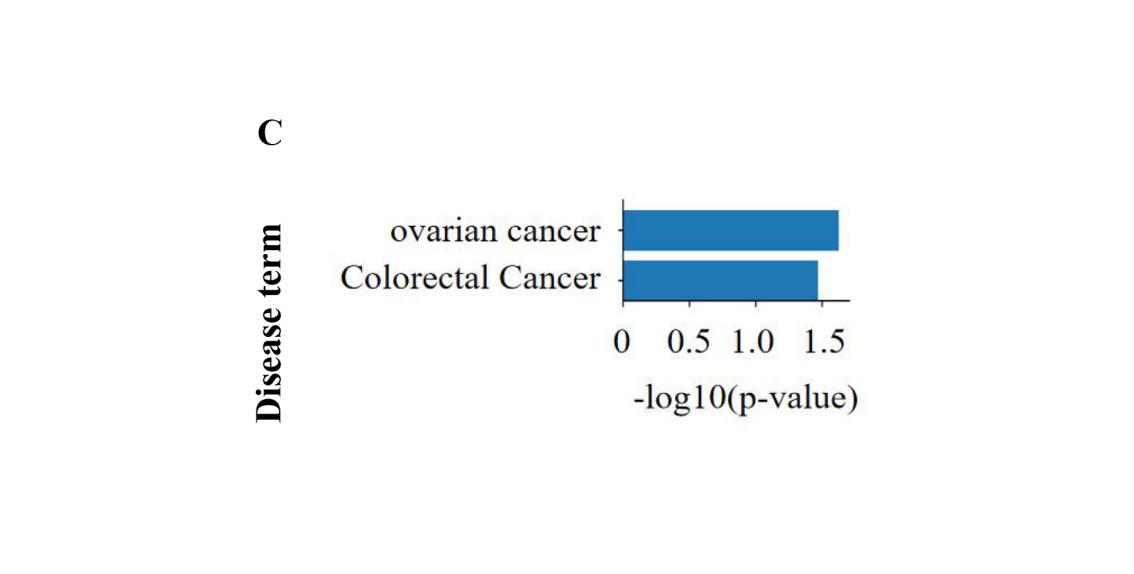


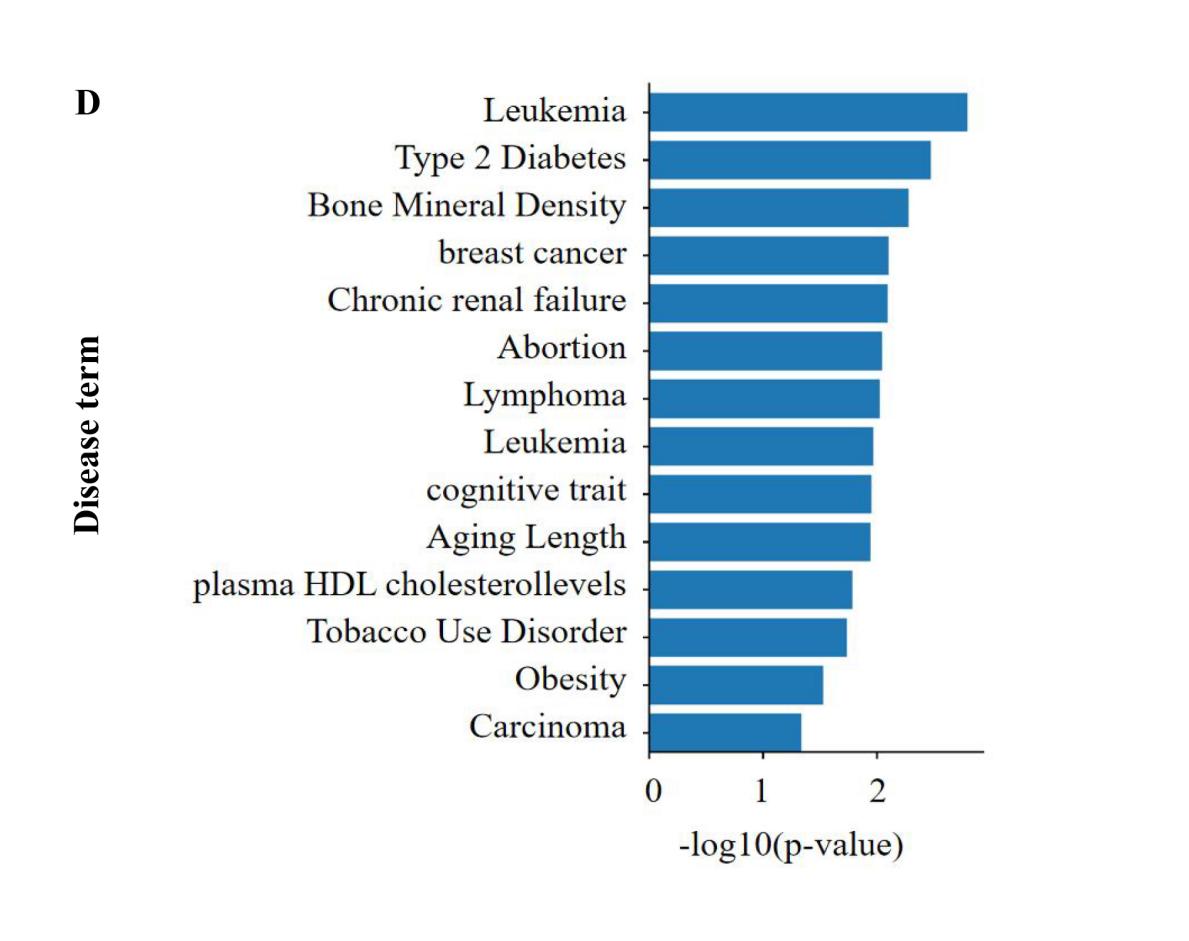


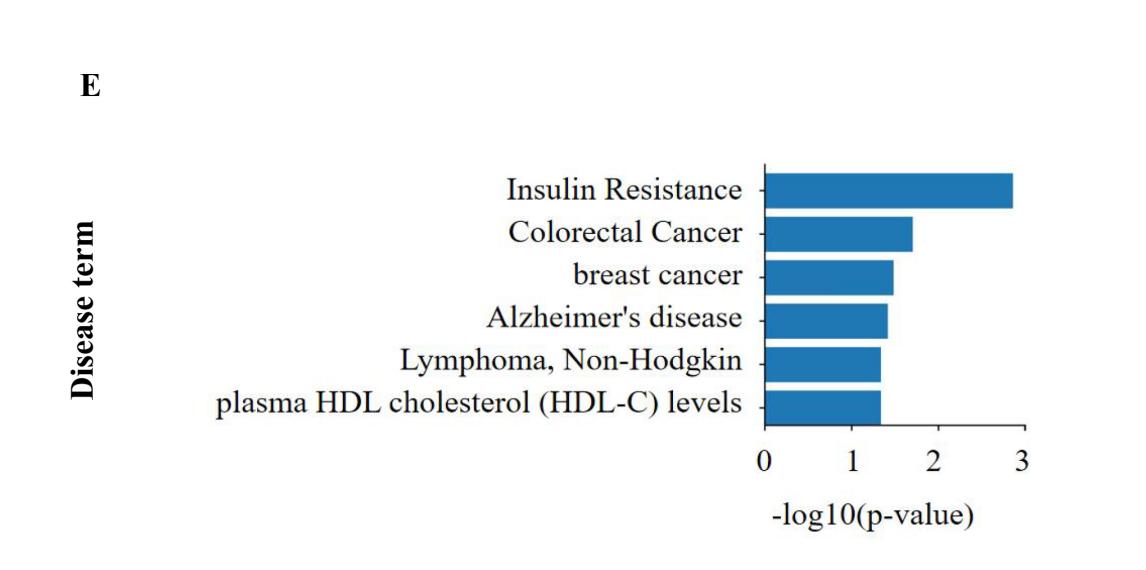


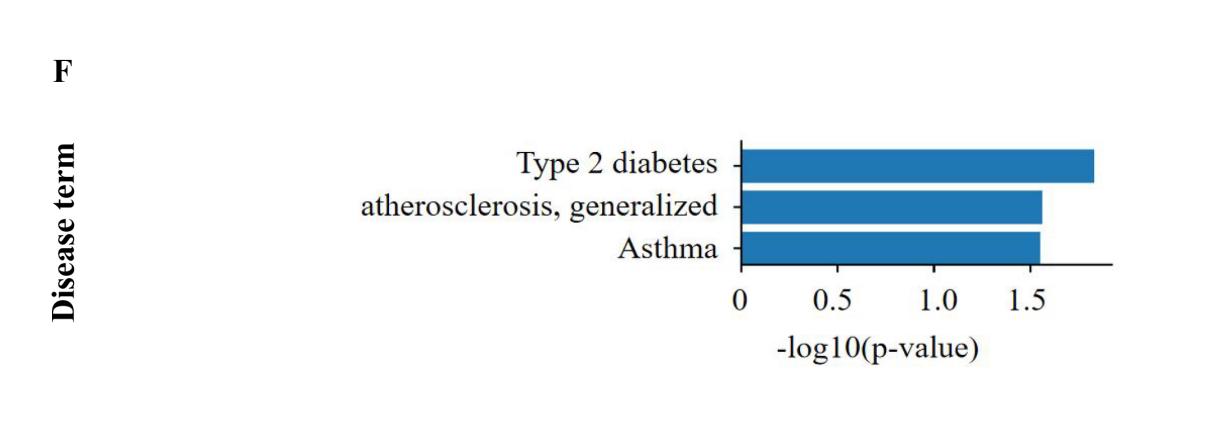


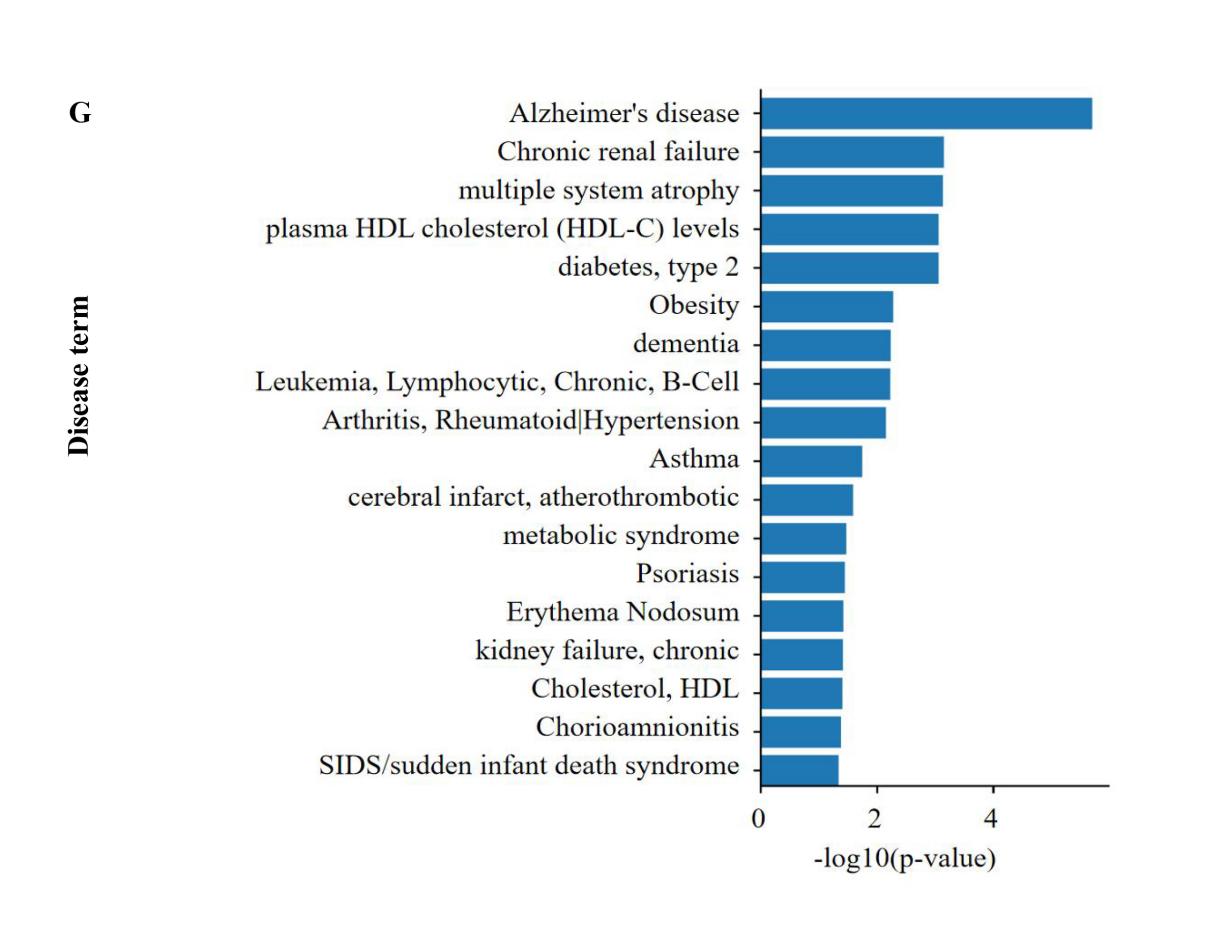


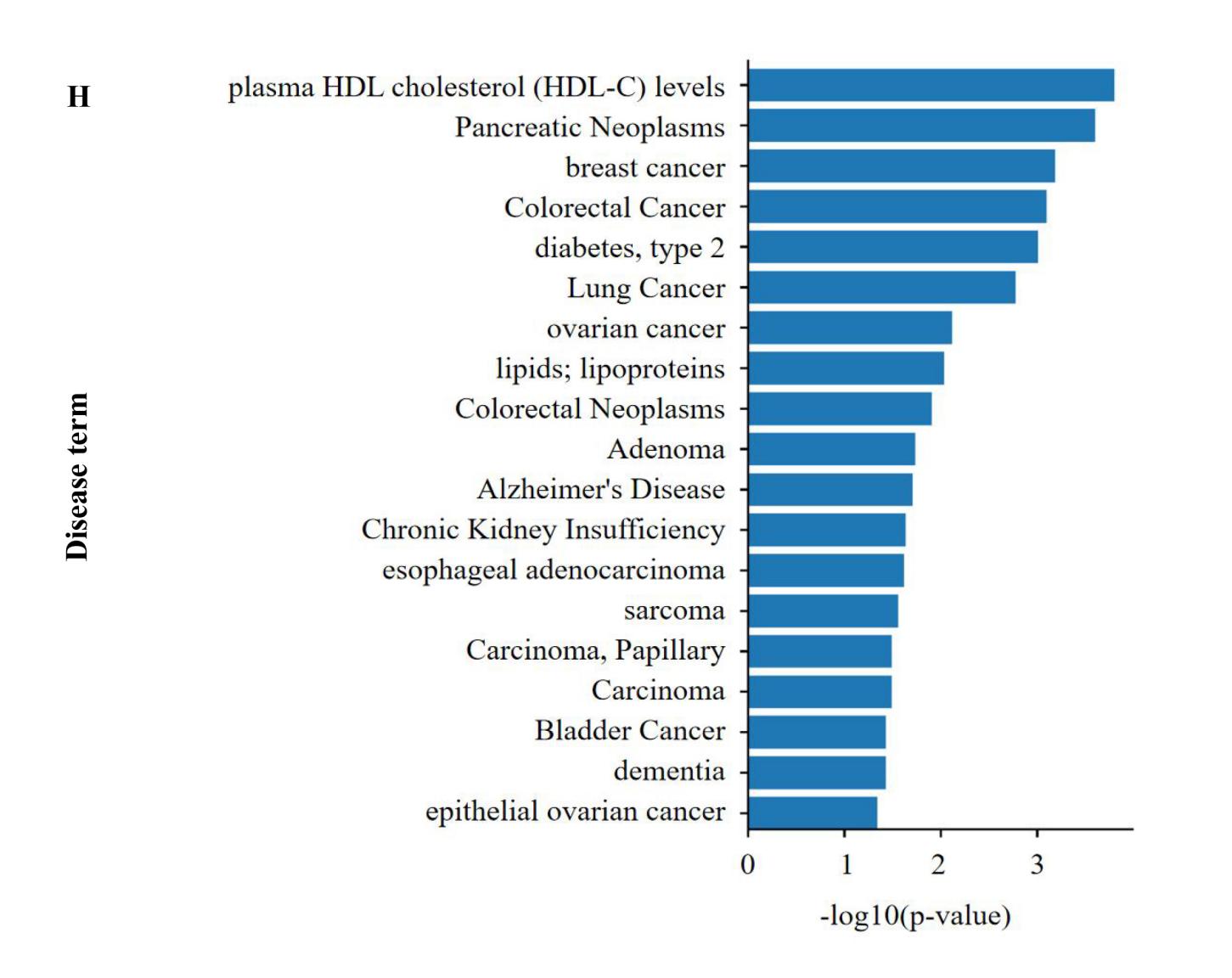


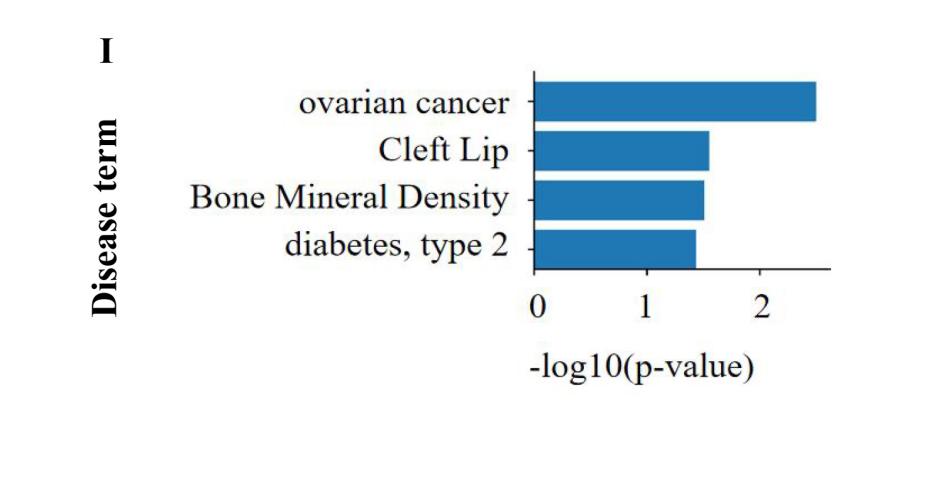


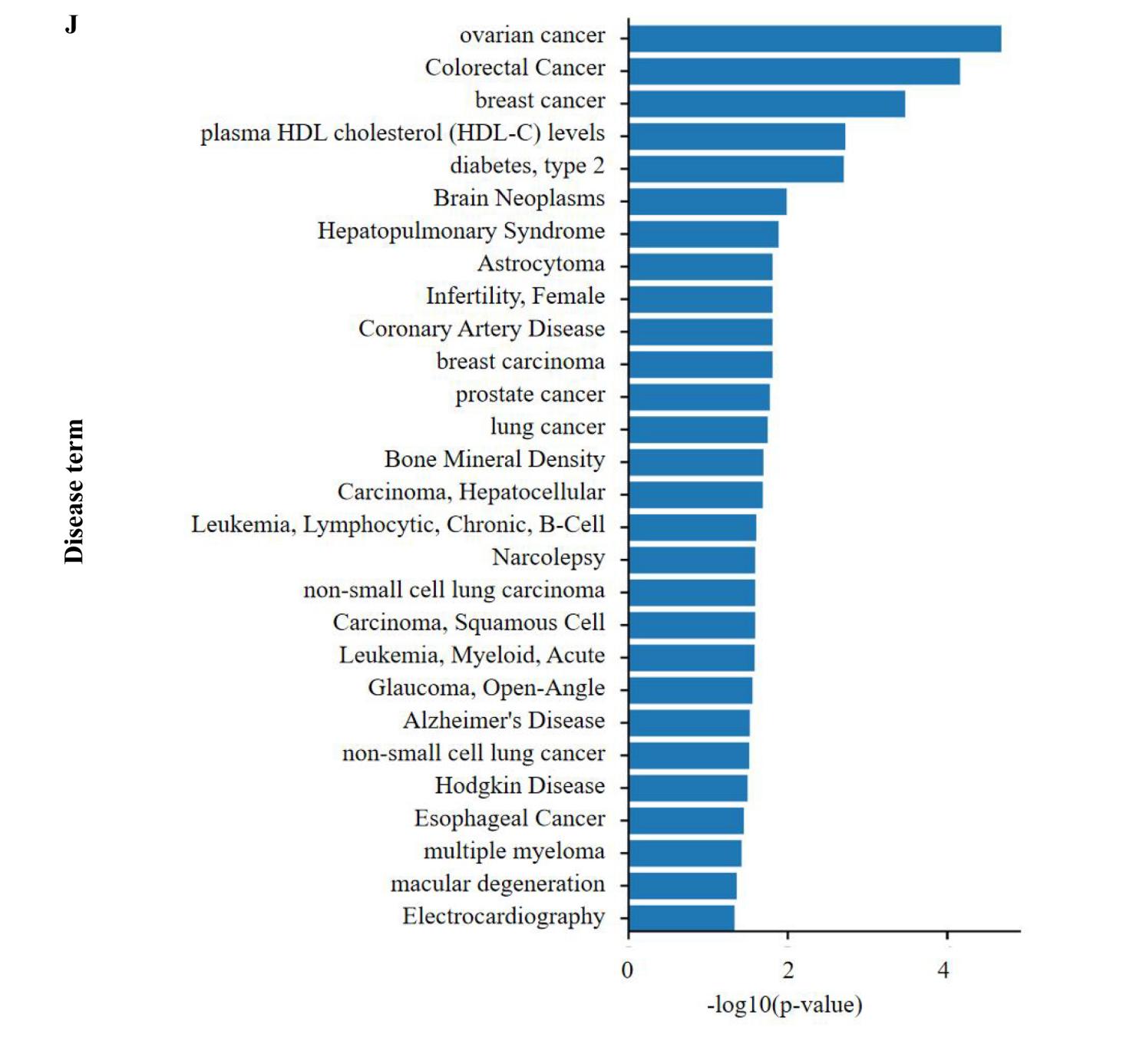

Supplement: Supplementary file 1 [file DataSheet1.ZIP › Supplementary_Material.docx]
